# Acquired genetic and cell-state changes in IDH-mutant glioma progression

**DOI:** 10.1038/s41586-026-10612-6

**Published:** 2026-06-03

**Authors:** Kevin C. Johnson, Avishay Spitzer, Frederick S. Varn, Masashi Nomura, Luciano Garofano, Tamrin Chowdhury, Anuja Lipsa, Linbin Zhang, Ester Calvo Fernández, Tanyeri Barak, A. Gulhan Ercan-Sencicek, Ayse Buket Peksen, Kevin J. Anderson, C. Mircea S. Tesileanu, Samirkumar B. Amin, Emre Kocakavuk, Dacheng Zhao, Fulvio D’Angelo, Simona Migliozzi, Lillian Bussema, Simon Gritsch, Hyo-Eun Moon, Sun Ha Paek, Franck Bielle, Alice Laurenge, Anna Luisa Di Stefano, Bertrand Mathon, Alberto Picca, Marc Sanson, Ann-Christin Hau, Frank Hertel, Kamil Grzyb, Zheng Zhao, Qianghu Wang, Tao Jiang, Julie J. Miller, Hiroaki Wakimoto, Daniel P. Cahill, Jennifer Moliterno, Murat Günel, Beth Hermes, Nader Sanai, Anna Golebiewska, Simone P. Niclou, Jason Huse, W. K. Alfred Yung, Anna Lasorella, Mario L. Suvà, Antonio Iavarone, Itay Tirosh, Roel G. W. Verhaak

**Affiliations:** 1 Department of Neurosurgery, Yale School of Medicine, New Haven, CT USA; 2 Department of Molecular Cell Biology, Weizmann Institute of Science, Rehovot, Israel; 3 Department of Oncology, Tel Aviv Sourasky Medical Center, Tel Aviv, Israel; 4 The Jackson Laboratory for Genomic Medicine, Farmington, CT USA; 5 Department of Genetics and Genome Sciences, University of Connecticut Health Center, Farmington, CT USA; 6 Institute for Systems Genomics, University of Connecticut, Storrs, CT USA; 7 Department of Pathology, Massachusetts General Hospital and Harvard Medical School, Boston, MA USA; 8 Broad Institute of MIT and Harvard, Cambridge, MA USA; 9 Department of Neurosurgery, Graduate School of Medicine, The University of Tokyo, Tokyo, Japan; 10 Krantz Family Center for Cancer Research, Massachusetts General Hospital and Harvard Medical School, Boston, MA USA; 11 Department of Public Health Sciences, Division of Biostatistics, University of Miami, Miller School of Medicine, Miami, FL USA; 12 Sylvester Comprehensive Cancer Center, University of Miami, Miller School of Medicine, Miami, FL USA; 13 NORLUX Neuro-Oncology Laboratory, Department of Cancer Research, Luxembourg Institute of Health, Luxembourg, Luxembourg; 14 Department of Hematology and Stem Cell Transplantation, West German Cancer Center (WTZ), National Center for Tumor Diseases (NCT) West, University Hospital Essen, University of Duisburg-Essen, Essen, Germany; 15 Department of Neurological Surgery, University of Miami, Miller School of Medicine, Miami, FL USA; 16 Department of Biochemistry and Molecular Biology, University of Miami, Miller School of Medicine, Miami, FL USA; 17 Department of Neurosurgery, Cancer Research Institute, Hypoxia Ischemia Disease Institute, Seoul National University, Seoul, Republic of Korea; 18 Advanced Institutes of Convergence Technology, Seoul National University, Gyeonggi-do, Republic of Korea; 19 Sorbonne Université, UMR S 1127, INSERM U 1127, CNRS, ICM–Paris Brain Institute, Equipe Labellisée LNCC, Paris, France; 20 AP-HP, Groupe Hospitalier Pitié-Salpêtrière, Neuropathology, Paris, France; 21 AP-HP, Groupe Hospitalier Pitié-Salpêtrière, Neuro-oncology, Paris, France; 22 Neurology Department, Foch Hospital, Suresnes, France; 23 Division of Neurosurgery, Azienda USL Toscana Nord-ovest, Livorno Hospital, Livorno, Italy; 24 AP-HP, Groupe Hospitalier Pitié-Salpêtrière, Neurosurgery, Paris, France; 25 Beijing Neurosurgical Institute, Capital Medical University, Beijing, China; 26 Chinese Glioma Genome Atlas Network & Asian Glioma Genome Atlas Network, Beijing, China; 27 Institute for Brain Tumors, Jiangsu Collaborative Innovation Center for Cancer Personalized Medicine, Nanjing Medical University, Nanjing, China; 28 Department of Bioinformatics, Nanjing Medical University, Nanjing, China; 29 Translational Neuro-Oncology Laboratory, Massachusetts General Hospital, Harvard Medical School, Boston, MA USA; 30 Stephen E. and Catherine Pappas Center for Neuro-Oncology, Department of Neurology, Massachusetts General Hospital, Harvard Medical School, Boston, MA USA; 31 Department of Neurosurgery, Massachusetts General Hospital, Harvard Medical School, Boston, MA USA; 32 St Joseph’s Hospital and Barrow Neurological Institute, Phoenix, AZ USA; 33 Ivy Brain Tumor Center at Barrow Neurological Institute, Phoenix, AZ USA; 34 Department of Pathology, The University of Texas MD Anderson Cancer Center, Houston, TX USA; 35 Department of Neuro-Oncology, The University of Texas MD Anderson Cancer Center, Houston, TX USA; 36 Department of Neurosurgery, Amsterdam University Medical Center, Amsterdam, The Netherlands; 37 Present Address: Translational Genomics Research Institute (TGen), Phoenix, AZ USA

**Keywords:** Oncogenesis, CNS cancer

## Abstract

Gliomas with mutant isocitrate dehydrogenase (IDH) are malignant brain tumours that typically arise in early to mid-adulthood and nearly always recur following treatment^1,2^. However, the genetic and cellular-state changes that drive IDH-mutant glioma progression under treatment remain incompletely understood. Here we integrated single-nucleus transcriptomic profiles, chromatin accessibility profiles and bulk DNA and RNA sequencing from 75 temporally separated gliomas across 35 patients comprising both the oligodendroglioma and astrocytoma IDH-mutant glioma tumour types. We show that malignant cell states transcriptionally resemble stages of normal glial–neuronal lineage development or a reactive mesenchymal-like state, mirroring states previously described in IDH wild-type glioblastoma^3,4^. Malignant cell states displayed distinct chromatin accessibility profiles that were comparable between both IDH-mutant glioma types. The abundance of less differentiated malignant cells increased with grade and with genetic alterations such as *PDGFRA* amplification. Longitudinal analysis highlighted two major malignant cell-state transition patterns. First, reduced lineage differentiation and increased proliferative malignant cells at recurrence were enriched in gliomas that acquired recurrence-associated genetic events. These included treatment-associated hypermutation, increased copy number changes and cell cycle alterations. Second, increased mesenchymal-like-state abundance occurred independently of acquired genetic alterations and instead coincided with elevated macrophage expression. Overall, our findings provide an integrative model that traces the cell intrinsic and extrinsic factors that shape cellular states during IDH-mutant glioma disease progression.

## Abstract (web summary)

Longitudinal transcriptomic, chromatin and genomic analyses of the two types of IDH-mutant glioma reveal in detail how disease progression is influenced by interdependent genetic, epigenetic and microenvironmental factors.

## Main

Hotspot mutations in the IDH genes *IDH1* and *IDH2* define a subset of adult-type diffuse gliomas with distinct molecular, histological and clinical features^1,2,5^. IDH-mutant gliomas are classified into two World Health Organization (WHO) tumour types: (1) oligodendroglioma, IDH-mutant and 1p/19q co-deleted (oligodendroglioma); and (2) astrocytoma, IDH-mutant (astrocytoma)^1,6^. Despite treatment with surgical resection, chemotherapy and radiotherapy, both oligodendroglioma and astrocytoma inevitably recur, which leads to substantial morbidity and mortality. Previous studies have suggested that therapeutic resistance may result from a combination of intratumoural cellular heterogeneity^4,7–10^, acquired genetic and epigenetic aberrations^9,11–13^ and a shift in myeloid cell populations^14^. A better understanding of the complex interplay among these molecular layers and how they influence the evolutionary paths of IDH-mutant gliomas is needed to guide the development of more effective therapeutic strategies.

To address these gaps, we aimed to establish a comprehensive portrait of treatment response and tumour evolution in IDH-mutant glioma through our Cellular Analysis of Resistance and Evolution (CARE) consortium. We profiled 75 longitudinally collected IDH-mutant glioma samples from 35 patients using single-nucleus RNA sequencing (snRNA-seq), complemented by matched bulk DNA sequencing (DNA-seq) and RNA sequencing (RNA-seq) and simultaneous single-nucleus chromatin accessibility (snATAC) profiling in a subset of samples. The integrated datasets enabled us to map the trajectories that IDH-mutant gliomas follow during disease progression and highlight how malignant cell states are shaped by epigenetics, genetics, microenvironment and therapy.

## CARE IDH-mutant cohort

**Fig. 1:**
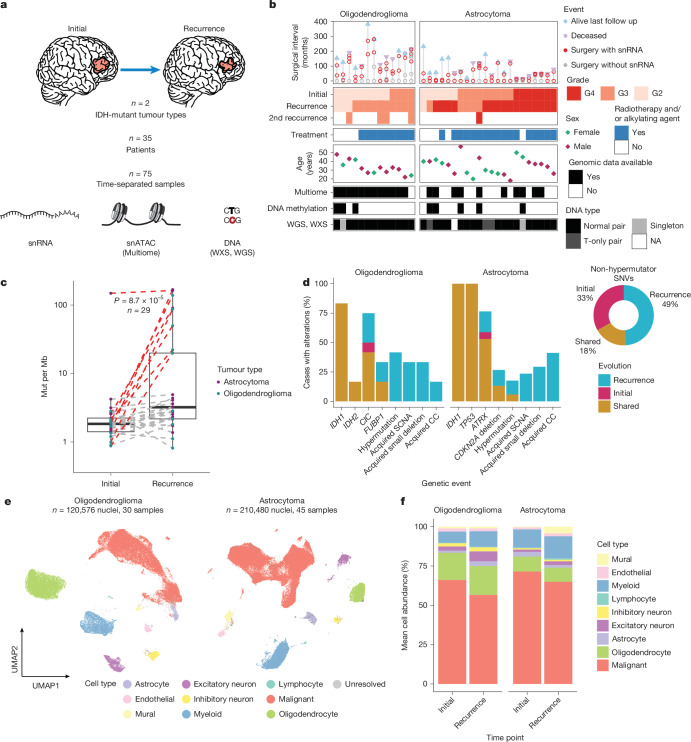
IDH-mutant longitudinal cohort and evolution under therapy. **a**, Schematic depicting the IDH-mutant glioma longitudinal cohort and molecular profiling approach. WXS, whole-exome sequencing. **b**, Cohort overview (*n* = 35 patients) stratified by WHO IDH-mutant glioma tumour type. Columns represent patients with longitudinal snRNA data. Top, timeline shows surgical interval and collection time points (months). Rows show tumour grade, treatment received between time points and age at diagnosis. Bottom bars show 10x Multiome, DNA methylation and DNA-seq data availability. **c**, Mutation burden, shown on the *y* axis, for matched initial and recurrent pairs with accompanying normal blood DNA samples. The box spans from the first to third quartiles, the median values are indicated by a horizontal line and whiskers show 1.5× the interquartile range. Dotted lines connect patient samples with one another, and red lines indicate hypermutated samples at the recurrence time point. Individual points are coloured by tumour type. Two-sided Wilcoxon signed-rank test is presented. **d**, Among patients with paired DNA-seq and matched normal blood samples (*n* = 29), the frequency of key genetic alterations, as determined by DNA-seq, is shown separately for oligodendroglioma and astrocytoma. Bars are coloured by the temporal pattern of each alteration: private to initial sample, shared across time points or private to recurrent sample. Highlighted events include canonical driver alterations and the changes associated with disease progression, including hypermutation, increased SCNA burden, increased small deletion burden and acquired cell cycle (CC) alterations. The doughnut chart represents the mean mutational proportion of shared and private mutations for patients without a hypermutation at recurrence. **e**, Uniform manifold approximation and projection (UMAP) plots for snRNA-seq data separated by tumour type. Individual nuclei are colour-coded by their cell-type annotation. **f**, The mean cell-type abundance is shown for each tumour type by time point. Colour represents the cell type. Schematic in **a** adapted from ref. ^8^, Springer Nature America.

**Extended Data Fig. 1:**
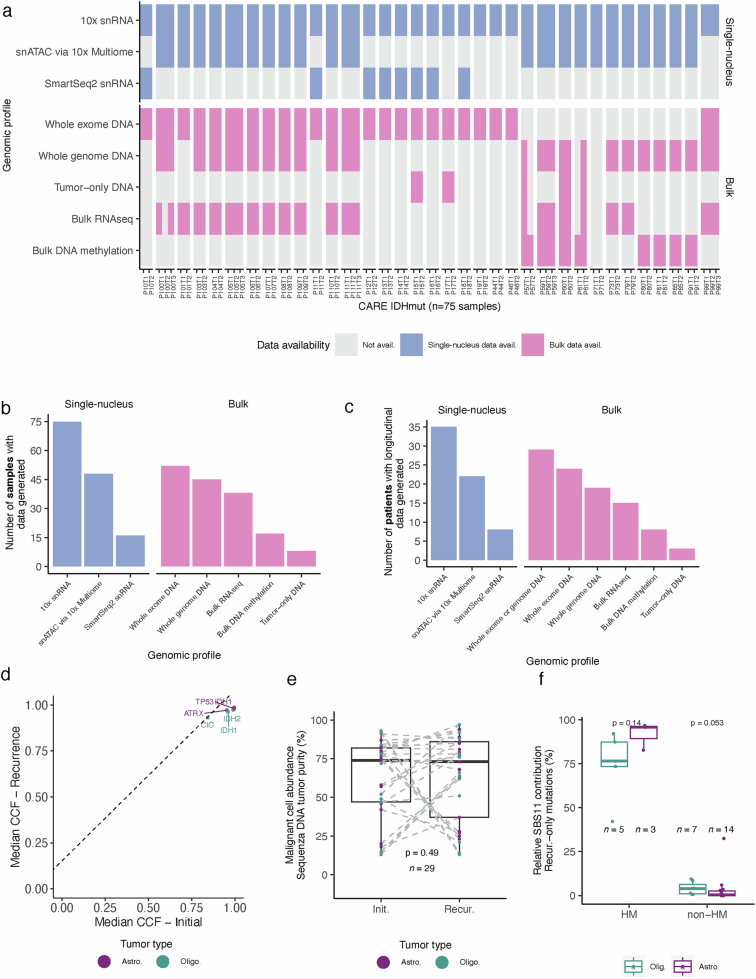
IDH-mutant glioma genomic cohort characteristics. (**a**) Grid layout indicating data availability for each sample included in this cohort (x-axis) with colors indicating data availability for single-nucleus or bulk sequencing approaches. (**b**) Cohort-level summary tallying the number of samples with profiling by each data type. (**c**) Cohort-level summary tallying the total number of longitudinal pairs with a data type available at matched initial and recurrent samples (**d**) Median PyClone estimated Cancer Cell Fraction (CCF) for selected driver genes for non-hypermutant longitudinal sample pairs. Values are presented separately for tumor type. (**e**) Matched initial and recurrent DNA purity estimates from Sequenza. For all box plots in this figure, the box spans from the first to third quartiles, median values are indicated by a horizontal line and whiskers show 1.5x the interquartile range. Patient samples are connected by a dotted line. Two-sided Wilcoxon signed-rank test P-value is shown is shown. (**f**) Relative COSMIC SBS11 mutational signature (alkylating agent-associated) proportion for all recurrence-only mutations are shown. Two-sided Wilcoxon rank-sum tests are shown comparing the tumor types. Analyses are separated for hypermutant samples (HM) and non-hypermutant samples (non-HM).

We collected longitudinal glioma samples from 35 patients with an IDH-mutant oligodendroglioma (*n* = 13) or an IDH-mutant astrocytoma (*n* = 22) diagnosis based on the 2021 WHO glioma classification at 2 or 3 time points^1^ (*n* = 75 samples; Supplementary Tables 1 and 2). For each patient, we designated the two earliest samples as the initial and recurrence for longitudinal analyses. In 17 out of 35 longitudinal pairs, the initial sample was obtained at primary diagnosis, whereas for the remaining 18 cases, the initial samples were collected at a subsequent surgery. Between the initial and recurrence samples, 26 out of 35 patients received radiotherapy and/or alkylating chemotherapy, whereas no adjuvant treatment was reported for the other patients. To comprehensively investigate IDH-mutant glioma evolution, we used snRNA-seq and bulk DNA-seq and RNA-seq from the same resected glioma samples (Fig. 1a,b). A subset of these samples was profiled with simultaneous snRNA–ATAC sequencing (48 out of 75), including 22 matched longitudinal pairs, to assess epigenetic changes. In parallel, a separate subset of samples (16 out of 75) was profiled by plate-based, full-length transcriptome Smart-seq2 (Extended Data Fig. 1a–c).

*IDH* mutations were longitudinally retained with a similar cancer cell fraction, and tumour purity was comparable at both time points (Extended Data Fig. 1d,e). We identified hypermutation associated with treatment with an alkylating agent (>10 mutations per Mb (mut per Mb)) with an enrichment for the SBS11 mutational signature (Fig. 1c and Extended Data Fig. 1f) in eight patients. Mutation burden was significantly increased at recurrence (*P* = 8.7 × 10^–5^, Wilcoxon signed-rank test; Fig. 1c), including when the analysis was restricted to samples that did not acquire a hypermutation (*P* = 0.01, Wilcoxon signed-rank test). We observed acquired genetic changes previously found to be enriched at recurrence^11,15,16^, including a >50% increase in somatic copy number alteration (SCNA) burden in ten patients, an increase in small deletion burden in four patients without hypermutation and cell cycle alterations (*CDKN2A* or* CDKN2B* homozygous deletion, *RB1* mutation or homozygous deletion, or *CDK4* amplification) in six patients who did not acquire hypermutation (Fig. 1d and Supplementary Fig. 1). Overall, 54% of the cohort acquired at least one of the following genetic alterations between the matched initial and recurrent samples: hypermutation, SCNA increase, small deletion increase or cell cycle alteration. All of these alterations were enriched among patients with a longitudinal increase in grade severity (*P* = 0.04, Fisher’s exact test).

## Longitudinal snRNA-seq profiles

**Extended Data Fig. 2:**
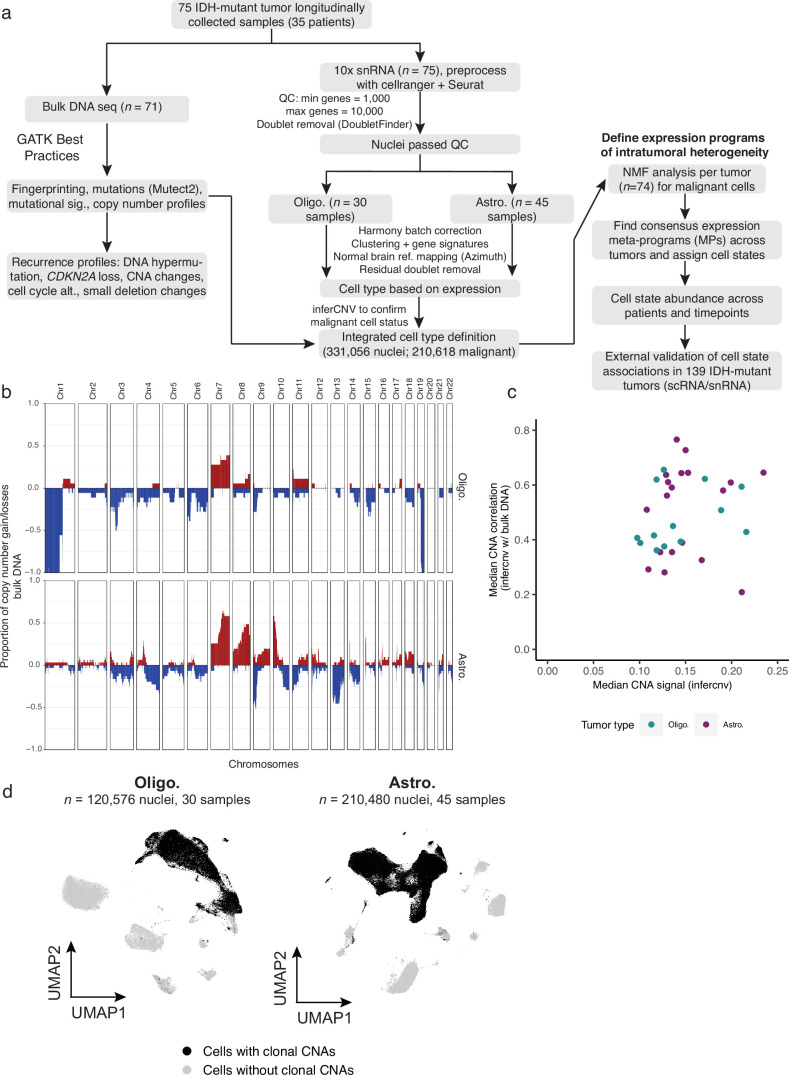
Integrated identification of malignant cell populations by copy number inference. (**a**) Analysis workflow for bulk DNA sequencing analysis and snRNA analysis including main quality control (QC) steps and analysis steps. (**b**) Per tumor type copy number alteration (CNA) frequency for bulk DNA samples passing CNA QC. Chromosomes in increasing order are presented on the x-ais. Positive (red) proportions reflect copy number gain frequency while negative (blue) proportions reflect copy number loss frequency. (**c**) For glioma samples that passed QC for both snRNA and bulk DNA CNA profiles, the median CNA signal (i.e., fraction of the measured genome with inferred CNA) is plotted against the median per chromosome Pearson’s correlation between CNA signal and bulk DNA CNA signal per patient. (**d**) UMAP plots presented in Fig. 1e are colored by whether CNA events consistent with malignancy (e.g., CNA loss at Chr1p) were detected in a cell (black) or not (gray).

**Extended Data Fig. 3:**
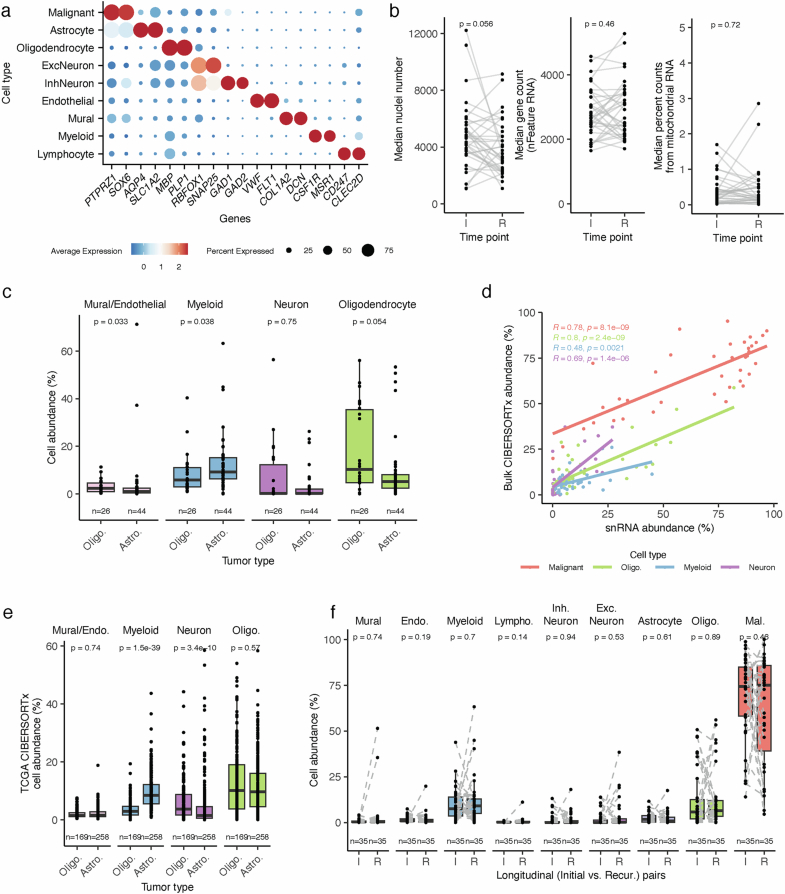
Tumor microenvironment differences across tumor types and time points. (**a**) Dot plot expression reflects the normalized and scaled gene expression of select marker genes per cell type across all samples (n = 75 samples). Two genes per cell type are shown. Color indicates average expression and size reflects the percentage of nuclei where that gene’s expression is detected. (**b**) Individual dots represent samples collected across time points (I = Initial, R = Recurrence, n = 35 patients) for QC metrics of nuclei number, median gene count, and median mitochondrial percentage counts. Lines connect two values for single patient. P-values represent a two-sided Wilcoxon signed-rank test. (**c**) Cell type or compartment abundance (i.e., combining abundance of similar cell types) are shown combining initial and recurrent samples from oligodendroglioma and astrocytoma samples. For all box plots in this figure, the box spans from the first to third quartiles, median values are indicated by a horizontal line and whiskers show 1.5x the interquartile range. P-values for represents two-sided Wilcoxon rank-sum test. (**d**) Two-sided Pearson’s correlation coefficients and associated P-values are shown for the correlation between snRNA cell type abundance and CIBERSORTx estimated abundance for samples where snRNA and bulk RNA were available for the same tumor (n = 38 CARE IDH-mutant samples). The four most abundant cell types are shown. (**e**) Estimated cell type abundance across TCGA oligodendroglioma and astrocytoma for selected cell types. (**f**) Longitudinal differences in microenvironment cell type abundance for matched initial (I) and recurrence (R, n = 35 patients) pairs. Dotted lines connect samples from the same patient. P-values represent two-sided Wilcoxon signed-rank test.

We assessed intertumoural and intratumoural cellular heterogeneity in the oligodendroglioma (*n* = 30) and astrocytoma (*n* = 45) samples using snRNA-seq. Overall, 331,056 single-nucleus transcriptomes passed quality control, with a mean of 3,096 genes detected (Extended Data Fig. 2a and Supplementary Table 3). Nuclei were clustered with Harmony batch correction and were annotated on the basis of marker gene expression, classification with a non-malignant brain atlas and the presence of inferred copy number alterations (CNAs) that were consistent with the bulk DNA profiles (Fig. 1e, Methods and Extended Data Fig. 2a–d). Nuclei annotated as malignant and lacking CNAs or nuclei annotated as non-malignant with CNAs were excluded (*n* = 4,636 nuclei), which produced a final set of 210,618 malignant and 115,802 non-malignant nuclei (Extended Data Fig. 2d). All non-malignant cell types were found in both tumour types and included oligodendrocytes, myeloid cells, endothelial cells, mural cells (that is, vascular smooth muscle cells and pericytes) and lymphocytes (largely T cells; Extended Data Fig. 3a). We also identified cell types often undetected in single-cell studies owing to technical bias, including excitatory neurons, inhibitory neurons and astrocytes. We did not observe significant differences in quality metrics between initial and recurrence samples (Extended Data Fig. 3b).

We observed a greater abundance of myeloid nuclei in astrocytoma samples (*P* = 0.04, Wilcoxon rank-sum test; Extended Data Fig. 3c). Oligodendroglioma samples contained a greater percentage of mural or endothelial nuclei, a result consistent with the branching network of capillaries in oligodendroglioma that define its ‘chicken wire’ histology^17^ (*P* = 0.03, Wilcoxon rank-sum test; Extended Data Fig. 3c). We validated the increase in myeloid abundance in astrocytomas by estimating cell-type abundance from bulk RNA-seq data from The Cancer Genome Atlas (TCGA) using CIBERSORTx deconvolution (Extended Data Fig. 3d,e). We did not observe a significant longitudinal shift in major cell-type abundances (Extended Data Fig. 3f).

## Malignant-state heterogeneity

**Fig. 2:**
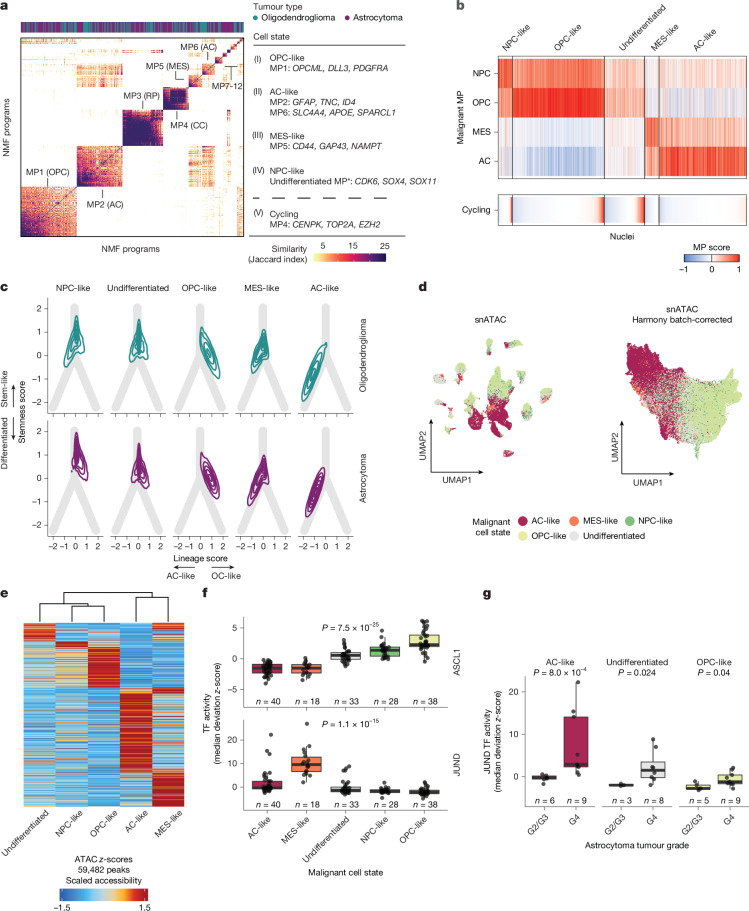
Malignant-cell-state heterogeneity and epigenetic regulation. **a**, Similarity matrix based on gene overlap (Jaccard index) for programs identified during the first round of metaprogram (MP) identification. The NMF programs are annotated by the tumour type of the sample. Each MP is identified by a number and a brief functional annotation, which are presented in full in Supplementary Table 4. The four annotated cell states (indicated by Roman numerals) and the cycling program are listed with key genes. The asterisk indicates the NPC-like MP, which was identified in the Undifferentiated population. RP, ribosomal gene program. **b**, MP scores per assigned cell state (*x* axis) across each MP (*y* axis) downsampled to 10,000 nuclei. The *x* axis is sorted by increasing cell cycle score. **c**, The density of each assigned state along a previously described state hierarchy^10^. Grey segments provide context for density. Positive stemness scores indicate greater stemness gene expression, whereas positive lineage scores reflect increased oligodendrocyte lineage expression. Negative lineage scores reflect increased astrocyte lineage expression. Colour reflects the tumour-type-specific density. **d**, UMAP plot for all malignant snATAC nuclei (*n* = 71,365) colour-coded by RNA cell state that is not batch-corrected (left) and Harmony batch-corrected UMAP (right). **e**, Heatmap for positive marker peaks per cell state (two-sided Wilcoxon rank-sum test, false discovery rate (FDR) < 0.05, log_2_[fold change] ≧ 1) is shown with hierarchal clustering. **f**, TF activity based on median chromVar deviation *z*-scores per cell state (minimum 20 nuclei, snATAC). Sample size indicates samples with at least 20 malignant nuclei per state. For all box plots in this figure, the box spans the first to third quartiles, the median values are indicated by a horizontal line and whiskers show 1.5× the interquartile range. **g**, Cell-state-specific median JUND activity across tumour grade in astrocytomas in non-MES-like nuclei. Significance was assessed by Kruskal–Wallis tests (**f**) or two-sided Wilcoxon rank-sum tests (**g**).

**Extended Data Fig. 4:**
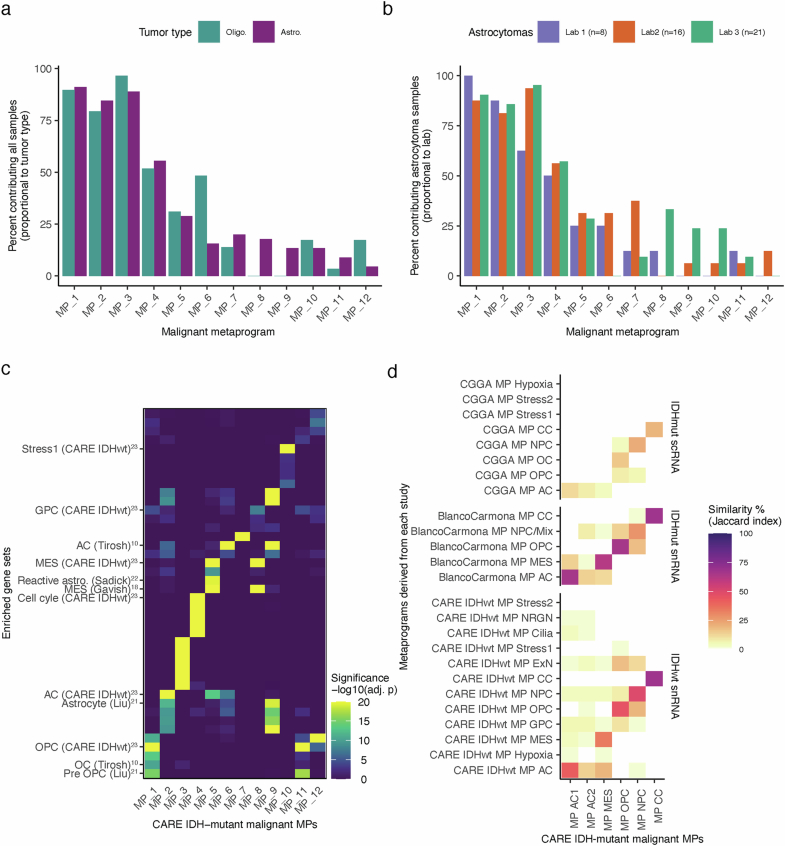
Malignant gene expression metaprograms. (**a**) The percentage of glioma samples that contributed to each of the 12 malignant metaprograms detected in this analysis per tumor type. (**b**) For astrocytomas, the only tumor type profiled across all laboratories, the percentage of astrocytoma samples that contributed to each of the 12 metaprograms detected in this analysis. (**c**) Functional enrichment heatmap for publicly available gene sets across metaprograms. Heatmap color represents the statistical significance for the enrichment and 5 gene sets are presented per metaprogram (one-sided hypergeometric test FDR adjusted P-value) with selected gene sets listed. (**d**) Heatmap reflects the similarity (Jaccard Index) between metaprogram (MP) gene sets derived in the present analysis and used for malignant cell state assignment (CARE IDHmut, x-axis), the CARE IDH-wildtype consortium (CARE IDHwt, snRNA, *n* = 121 samples)^23^, Blanco-Carmona et al. (IDH-mutant, snRNA, *n* = 26 samples)^25^, and samples profiled by the Chinese Glioma Genome Atlas (CGGA, IDH-mutant scRNA, *n* = 43 samples)^26^.

Next, we applied a framework based on non-negative matrix factorization (NMF) to derive malignant gene expression metaprograms that vary within glioma samples and across multiple glioma samples^18–20^ (Fig. 2a). This analysis identified 12 metaprograms that represented patterns of intratumoural heterogeneity and were repeatedly observed across samples. These were refined to five by excluding batch-associated or quality-associated metaprograms and requiring detection in both tumour types (Fig. 2a, Extended Data Fig. 4a,b and Supplementary Table 4).

To annotate each metaprogram, we examined previous malignant and non-malignant brain gene sets^4,10,18,21,22^ (Extended Data Fig. 4c). These analyses identified metaprograms enriched for the previously reported oligodendrocyte and oligodendrocyte progenitor cell-like (OC/OPC-like), astrocyte-like (AC-like) and cell cycle signatures. This analysis also revealed a previously undescribed IDH-mutant metaprogram that was enriched for gene sets including an IDH wild-type mesenchymal-like (MES-like, hypoxia independent) state and a non-malignant reactive astrocyte state^3,10,22,23^ (Extended Data Fig. 4c). Thus, we annotated the five malignant nuclei metaprograms as follows: OC/OPC-like (shortened to OPC-like, MP1); AC-like (MP2 and MP6); MES-like (MP5); and a cycling state (MP4). Next, we assigned nuclei to OPC-like, AC-like or MES-like states on the basis of whether the relative expression score for each state was significantly different from a null distribution of shuffled gene expression. Nuclei that were not confidently classified into a state were assigned to an ‘Undifferentiated’ state that reflected their lack of commitment to differentiated cell programs. This approach was repeated independently for the cell cycle such that a nucleus could be classified into one of the three states as well as whether it was cycling. Notably, the same metaprograms were detected when restricting the analyses to only oligodendrogliomas or astrocytomas.

We reasoned that the Undifferentiated-state population might contain multiple cell states that each exist at low frequency and may be challenging to identify when analysed among all malignant nuclei. We repeated the NMF approach using only the Undifferentiated state population, seeking to identify subsets of cell states in this population. This analysis revealed a single novel metaprogram that demonstrated high gene overlap with the IDH wild-type neural progenitor cell (NPC)-like state^3,23^ (Extended Data Fig. 4d). Other metaprograms resembled OPC-like and AC-like states, a result consistent with the weak expression of the astrocytic and oligodendrocyte lineage programs. Thus, the Undifferentiated state represents a combination of rare states, including more primitive nuclei that can be classified into an NPC-like state and nuclei without a well-defined expression program, a finding consistent with previous observations^10,24^. To further validate IDH-mutant states, we applied our NMF-based framework to an external IDH-mutant glioma snRNA dataset (*n* = 26 samples) and an external IDH-mutant glioma single-cell RNA (scRNA) dataset (*n* = 43 samples)^25,26^. Although different metaprogram genes were detected across single-nucleus and single-cell technologies, the analysis independently identified the functional malignant states we defined (Extended Data Fig. 4d). Finally, we added the NPC metaprogram to our classification and re-assigned nuclei to NPC-like, OPC-like, AC-like, MES-like or Undifferentiated states for all downstream analyses.

**Extended Data Fig. 5:**
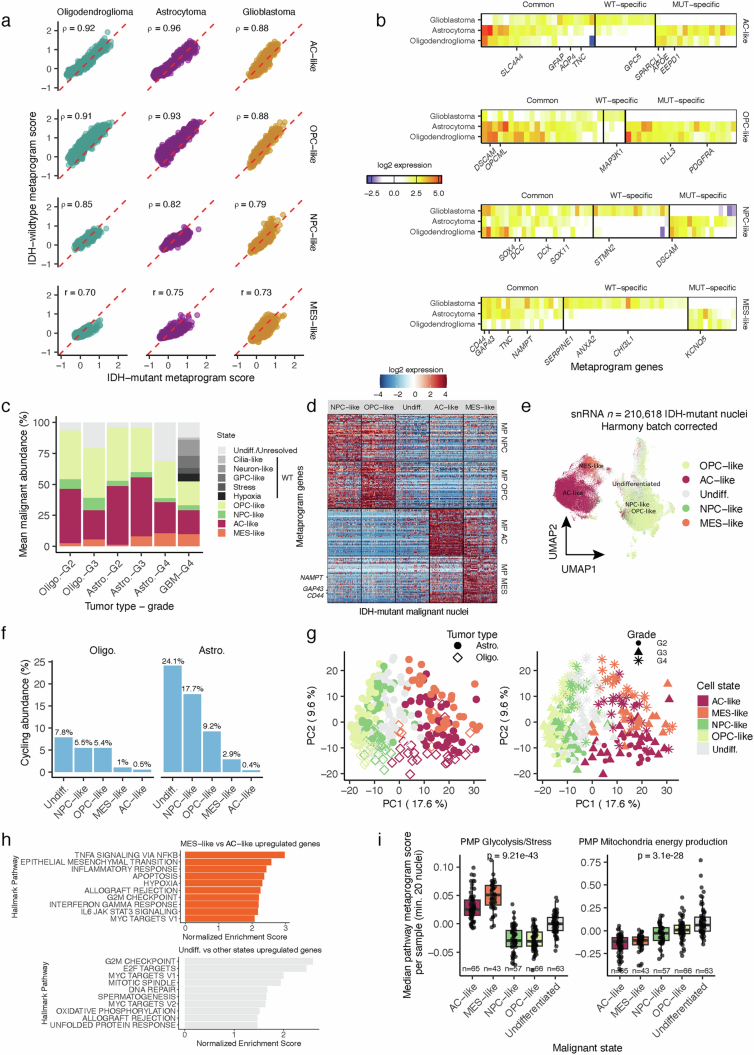
Malignant states display variable features of differentiation, proliferation, and metabolism. (**a**) Malignant nuclei from CARE IDH-wildtype (Glioblastoma, GBM)^23^ and IDH-mutant (present study) cohorts were scored for both IDH-mutant metaprograms (MPs, x-axis) and IDH-wildtype MPs (y-axis). Spearman correlation coefficient is shown and dotted red line represents a slope of 1. Row facets represent glioma metaprograms shared across tumor types. Columns represent glioma tumor types. (**b**) Relative gene expression of AC-like, OPC-like, NPC-like, and MES-like malignant nuclei in each glioma tumor type (rows), with genes (columns) expressed in that metaprogram that are common to IDH-mutant and IDH-wildtype or specific. (**c**) Relative malignant cell abundance for across different tumor types and grades, including CARE GBM samples (*n* = 121). Additional states defined in IDH-wildtype are indicated. (**d**) Heatmap representing the relative gene expression for each metaprogram gene (y-axis) and nuclei assigned to a state (x-axis). Selected genes are shown for the indicated row. Each cell state was down sampled to 200 nuclei. (**e**) Harmony batch-corrected UMAP for all malignant nuclei colored by state (**f**) Bar plot indicates the relative proportion of each state that is cycling for both oligodendrogliomas and astrocytomas. (**g**) Principal components analysis (PCA) plots for pseudobulk gene expression of each state per sample (minimum 20 nuclei per state). The pseudobulk profiles are colored by cell state and shape indicates tumor type (left) or tumor grade (right). (**h**) Gene set enrichment analysis for genes upregulated in pseudobulk MES-like compared with AC-like (top) and for genes upregulated in pseudobulk Undifferentiated compared with all other malignant states (bottom). (**i**) Median malignant cell scores per sample for selected pathway-based metaprograms (PMPs). Selected PMPs reflect difference in metabolic pathway utilization across malignant states. *P*-values reflect Kruskal-Wallis test.

The consistency in malignant programs across IDH wild-type^3,23^ and IDH-mutant gliomas (Extended Data Fig. 4d), despite their distinct genetic and clinical features, suggests that there are core malignant states shared across glioma types. To test this idea, we compared malignant transcriptomes across all glioma types and observed strong positive correlations in metaprogram expression (for example, Spearman’s *ρ* = 0.88–0.96 for AC/OPC-like states; Extended Data Fig. 5a). Nevertheless, expression differences specific to tumour types showed that IDH-mutant and IDH wild-type gliomas rely on distinct transcriptional networks to give rise to these states, with variations in state abundance across glioma tumour types (Extended Data Fig. 5b,c). Together, these analyses highlight the shared lineage and reactive states across gliomas and define the transcriptional features that distinguish tumour types.

We next compared the metaprogram scores across nuclei. We observed that the MES-like state shares expression features with the AC-like state but has higher expression of IDH wild-type MES-like genes (*CD44*, *NAMPT* and *GAP43*). Together with the clustering data, this result suggests that the MES-like state reflects a more granular classification of AC-like nuclei (Fig. 2b and Extended Data Fig. 5d,e). This analysis also revealed that the Undifferentiated state had the highest cycling percentage (7.8% for oligodendrogliomas and 24.1% for astrocytomas), followed by NPC-like, OPC-like and MES-like, whereas <1% of AC-like nuclei were classified as cycling (Fig. 2b and Extended Data Fig. 5f). This result is consistent with a negative relationship between differentiation and proliferation^4^. Finally, we scored these nuclei along the cellular hierarchy proposed by our earlier studies^10^, which highlighted the intermediate and progenitor transcriptional profile of the Undifferentiated state and confirmed that there are more frequent cycling populations near the apex of the hierarchy (Fig. 2c and Supplementary Fig. 2).

To further support these transcriptionally distinct states, we constructed pseudobulk profiles for each state per sample and performed principal component analysis (PCA). Principal component 1 (PC1) separated pseudobulk profiles by malignant state independent of tumour type, which highlighted the shared lineages across IDH-mutant glioma. By contrast, PC2 stratified pseudobulk profiles by tumour type and grade (Extended Data Fig. 5g). Pseudobulk differential expression analysis supported an upregulation of inflammatory responses in the MES-like state compared with the AC-like state. Conversely, Undifferentiated malignant nuclei compared with all other malignant states had upregulated genes that were enriched for cell cycle and oxidative phosphorylation pathways (Extended Data Fig. 5h). We confirmed the upregulation of mitochondrial energy production pathways in the stem-like states, such as Undifferentiated, and the greater glycolytic pathway use in AC-like and MES-like nuclei. This result highlights the metabolic differences across these states (Extended Data Fig. 5i). Collectively, our analyses identified five consensus IDH-mutant malignant states that differ in lineage differentiation, proliferation and metabolism.

## Epigenetic regulation of cell states

**Extended Data Fig. 6:**
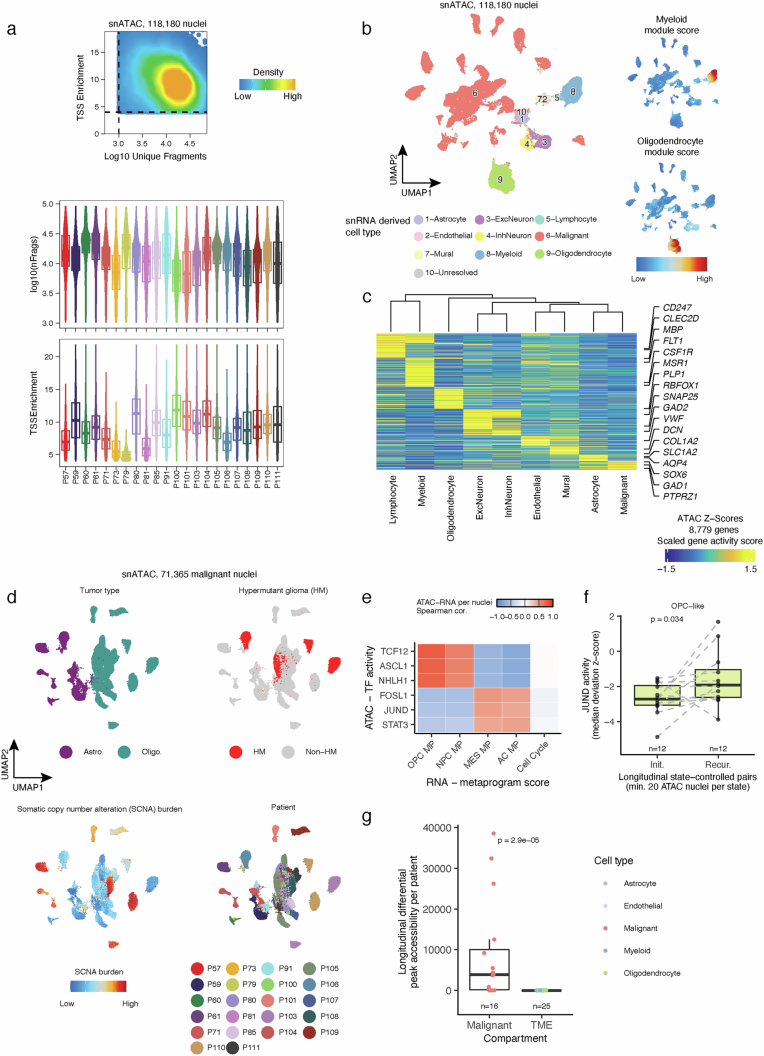
Microenvironmental and malignant cell chromatin accessibility. (**a**) Top: The density for the number of unique ATAC fragments per nuclei are plotted against the transcriptional start site (TSS) enrichment score for all snATAC data (118 K nuclei). Bottom: Box and violin plots per patient reflect the number of fragments and TSS enrichment scores. For all box plots in this figure, the box spans from the first to third quartiles, median values are indicated by a horizontal line and whiskers show 1.5x the interquartile range. (**b**) UMAP plot for snATAC Multiome data annotated by snRNA-based cell type (left). The same UMAP plot is shown on the right colored by the gene accessibility module score (snATAC) for the snRNA cell-type specific marker genes for myeloid nuclei (top right) and oligodendrocytes (bottom right). Red indicates high relative chromatin accessibility for the RNA-based cell type marker genes. (**c**) Gene accessibility score heatmap for cell type marker genes identified in snATAC data (FDR < 0.05, log2FC >= 1). Cell types are clustered based on marker gene accessibility. Selected genes are presented as row annotation. (**d**) All malignant nuclei snATAC UMAP color-coded by tumor type (top left), hypermutation status (top right), somatic copy number alteration burden (infercnv, bottom left), and by patient (bottom right), (**e**) Spearman’s correlation coefficient between per nuclei RNA metaprogram expression (snRNA) and transcription factor (TF) activity based on chromVar deviation z-scores (snATAC) is shown for selected cell state regulators. (**f**) Cell state-specific median JUND activity (chromVar, snATAC) across time points for a non-MES-like state. P-value represents two-sided Wilcoxon signed-rank test. Dotted lines connect patient samples across time points. (**g**) Per patient, longitudinal cell state-specific differentially accessible peaks across malignant and tumor microenvironmental cell types. For each cell state and patient, at least 50 nuclei were required for both time points. Individual data points are shown. P-value represents two-sided Wilcoxon rank-sum test.

To evaluate the epigenetic features of these transcriptionally distinct states, we analysed nuclei with simultaneous snATAC profiles, retaining nuclei that passed both RNA and ATAC quality control metrics (118,180 total nuclei and 71,365 malignant nuclei; Extended Data Fig. 6a). Unsupervised clustering of snATAC data demonstrated 99% cell-type assignment concordance between the two platforms. Moreover, cell-type-specific gene accessibility was consistent with gene expression differences (Extended Data Fig. 6b,c). ATAC-based clustering of malignant nuclei revealed the impact of malignant state, tumour type (that is, 1p/19q co-deletion), hypermutation and SCNA burden on chromatin accessibility profiles (Fig. 2d, left, and Extended Data Fig. 6d). Applying Harmony batch correction to mediate the sample-specific differences in genetics highlighted alignment with transcriptional states (Fig. 2d, right). Transcriptional state-specific differentially accessible peaks and genes further supported epigenetic differences between the more stem-like (NPC-like, OPC-like and Undifferentiated) and more differentiated (AC-like and MES-like) populations (Fig. 2e and Supplementary Fig. 3).

To identify key cell-state transcriptional regulators, we examined enrichment of transcription factor (TF) motifs among differentially accessible chromatin peaks and estimated the per-cell TF activity on the basis of genome-wide motif accessibility. We then filtered the set of TFs to those for which inferred activity showed a positive correlation with metaprogram gene expression (Spearman’s *ρ* > 0.4). This analysis revealed that basic helix-loop-helix TFs important in neurogenesis, such as TCF12 and ASCL1, were active in more stem-like states^27,28^ (Fig. 2f and Extended Data Fig. 6e). By contrast, TFs with the greatest activities in more differentiated malignant nuclei included members of the activator protein 1 complex (AP-1, JUN and FOS) and STAT3, which have previously been associated with injury and stress responses and the IDH wild-type MES-like state^29–31^. Although most tightly linked with the MES-like state, we observed increased activity in AP-1 members (for example, JUND) in non-MES-like nuclei with increasing grade (Wilcoxon rank-sum test, *P* = 8.0 × 10^–4^, *P* = 0.02, *P* = 0.04; Fig. 2g). Similarly, JUND (AP-1) activity increased in matched recurrent samples among non-MES-like cell states, which suggested that there is a chromatin state shift at advanced disease (Wilcoxon signed-rank test, *P* = 0.03; Extended Data Fig. 6f). Longitudinal differences in chromatin accessibility peaks were significantly greater in malignant than in non-malignant nuclei, which provides further support for the occurrence of epigenetic reorganization in the malignant compartment (Wilcoxon rank-sum test, *P* = 2.9 × 10^–5^; Extended Data Fig. 6g). These results suggest that IDH-mutant glioma states are epigenetically encoded and identify key regulators that govern transcriptional changes during disease progression.

## Reduced differentiation with progression

**Fig. 3:**
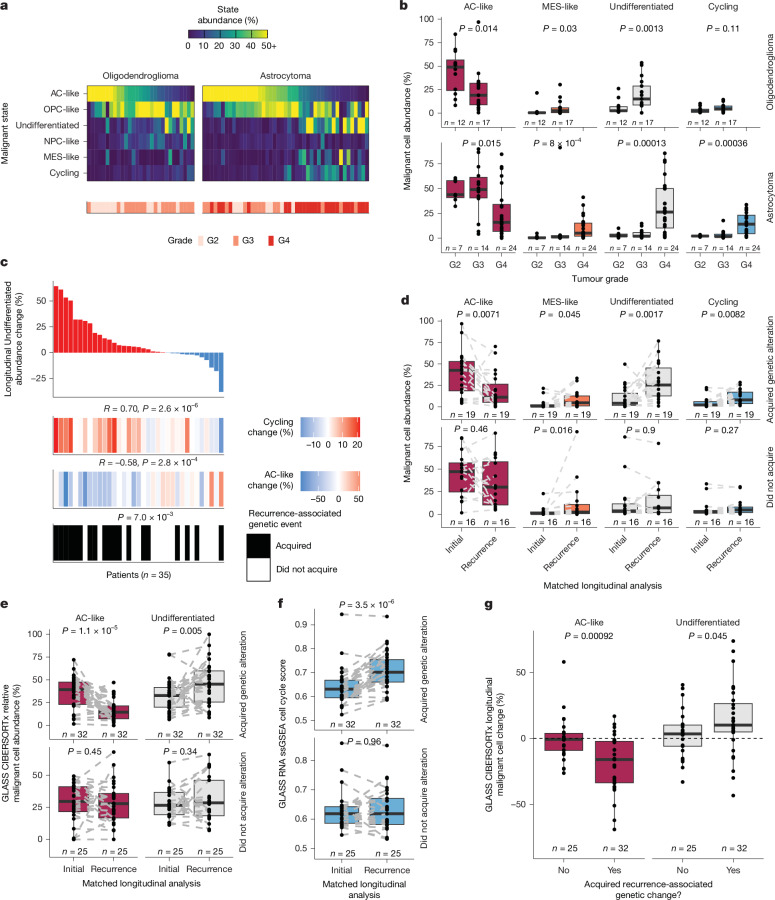
Malignant-state architecture is shaped by grade and genetic changes. **a**, Heatmap reflecting the relative malignant-state abundance for all cell states ordered by decreasing AC-like abundance for samples with at least 100 malignant nuclei (*n* = 74). Each sample is an individual column, and the annotation bar at the bottom indicates the tumour grade. The percentage of all malignant nuclei that are cycling is shown at the bottom of the malignant-state features. **b**, Per-sample malignant-cell-state abundance separated by tumour type and tumour grade (for example, grade 2 = G2). For all box plots in this figure, the box spans from the first to third quartiles, the median values are indicated by a horizontal line and whiskers show 1.5× the interquartile range. For oligodendroglioma, *P* values reflect two-sided Wilcoxon rank-sum tests, whereas *P* values for astrocytomas reflect Kruskal–Wallis tests. **c**, Waterfall plot depicting the longitudinal change (recurrence – initial) in Undifferentiated state abundance. Red indicates increase, blue indicates decrease. Each column indicates a patient (*x* axis). Annotation bars are presented for cycling and AC-like changes in these same samples. Two-sided Pearson’s correlation coefficients and associated *P* values are shown. Bottom, *P *value representing two-sided Wilcoxon rank-sum tests comparing differences in longitudinal Undifferentiated abundance between those with acquired recurrence-associated genetic alterations and those without. **d**, Longitudinal changes in malignant-cell-state abundance for selected states. The panel is split by samples with acquired genetic alterations (top) and more genetically stable samples (bottom). *P* values represent two-sided Wilcoxon signed-rank tests. **e**,**f**, Same framework as **d** with estimated CIBERSORTx fractions (**e**) and single-sample gene set enrichment analysis (ssGSEA) scores (**f**) for DNA and RNA data from the GLASS cohort (*n* = 57 longitudinal patients). **g**, Longitudinal differences between glioma samples that acquired a recurrence-associated genetic alteration (*n* = 32) versus samples that did not (n = 25). *P* value represents a two-sided Wilcoxon rank-sum test.

**Extended Data Fig. 7:**
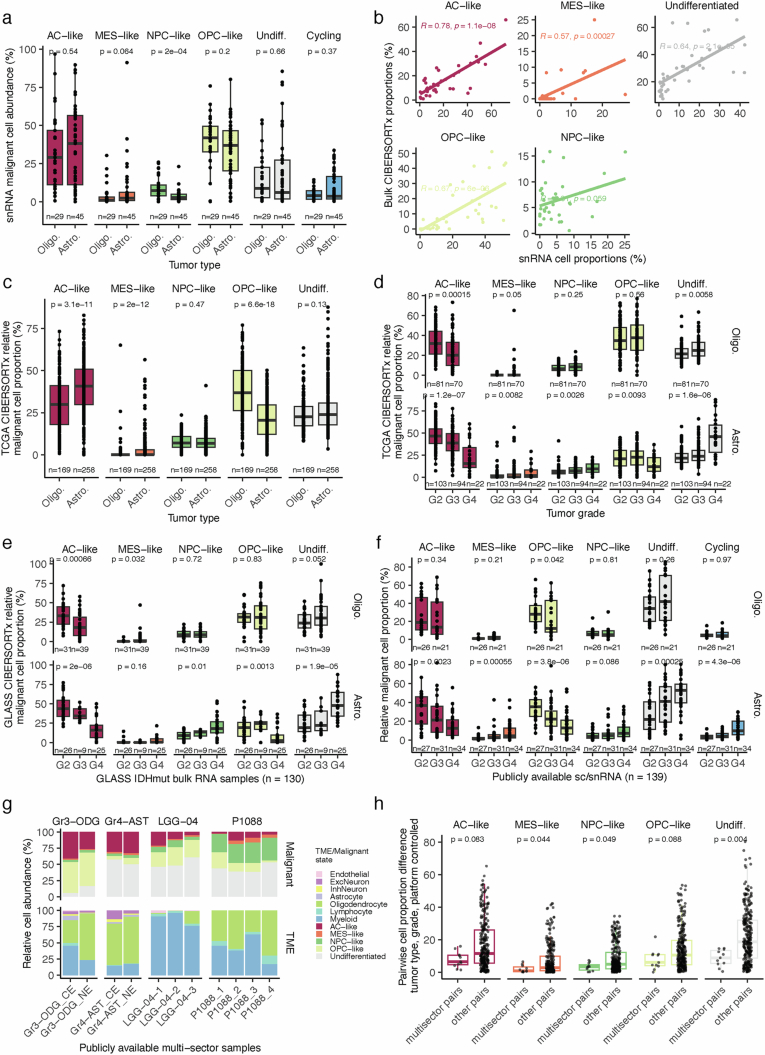
Malignant cell state abundance across glioma tumor type, grade, and region. (**a**) Malignant cell abundance across tumor types. For all box plots in this figure, the box spans from the first to third quartiles, median values are indicated by a horizontal line and whiskers show 1.5x the interquartile range. P-values comparing two groups indicate two-sided Wilcoxon rank-sum test and comparisons across three groups indicate Kruskal-Wallis tests. (**b**) snRNA malignant cell state proportions are plotted against CIBERSORTx inferred malignant proportions (bulk RNA) collected from the same samples (n = 37). Two-sided Pearson’s correlation coefficient and associated P-value are presented. (**c**) TCGA CIBERSORTx estimated cell state abundances across tumor types. (**d**) TCGA CIBERSORTx estimated cell state abundance across tumor grade for oligendroglioma and astrocytoma. (**e**) GLASS CIBERSORTx estimated malignant cell state abundance is shown across tumor grade in oligodendroglioma and astrocytoma. (**f**) Malignant state abundance for publicly available sc/snRNA IDH-mutant glioma data (n = 139) is compared across grade for oligodendroglioma and astrocytoma. (**g**) Malignant (top) and tumor microenvironment (bottom) cell state abundance for sc/snRNA are plotted for external cohort samples that were collected from different regions of the same tumor including samples from contrast enhancing (CE) and non-enhancing (NE) regions or unannotated regions of the tumor (i.e., numbered 1-4). (**h**) Pairwise cell state abundance differences calculated for samples collected from the same tumor (i.e., multisector pairs, left) and those calculated between samples from different patients controlled for tumor type, grade, and platform (i.e., other pairs).

We investigated whether malignant-state abundance differed by tumour type and tumour grade. Although largely comparable, we found increased NPC-like-state abundance in oligodendrogliomas (Wilcoxon rank-sum test, *P* = 2.0 × 10^–4^; Extended Data Fig. 7a). We estimated malignant-state-cell abundance in TCGA using CIBERSORTx (Extended Data Fig. 7b,c), which highlighted that state distributions were skewed depending on the tumour type. We next focused on tumour grade and observed a significant decrease in the differentiated AC-like cell fraction and an increase in the MES-like, Undifferentiated and cycling fractions with increasing grade across tumour types (Fig. 3a,b). We validated these grade-associated findings both in bulk RNA deconvolution data from the TCGA (*n* = 370) and the Glioma Longitudinal Analysis (GLASS) consortium (*n* = 130), and in external IDH-mutant scRNA and snRNA datasets (*n* = 139; Extended Data Fig. 7d–f and Supplementary Table 5). To evaluate how spatial heterogeneity affects malignant-cell-state distribution, we re-analysed external scRNA and snRNA data collected from spatially distinct tumour regions (*n* = 11 samples) and observed that malignant-state composition was comparatively stable across regions^32,33^ (Extended Data Fig. 7g,h). Together, these results support and extend our earlier observations regarding grade-related changes towards reduced differentiation and increased MES-like abundance with increasing tumour grade.

**Extended Data Fig. 8:**
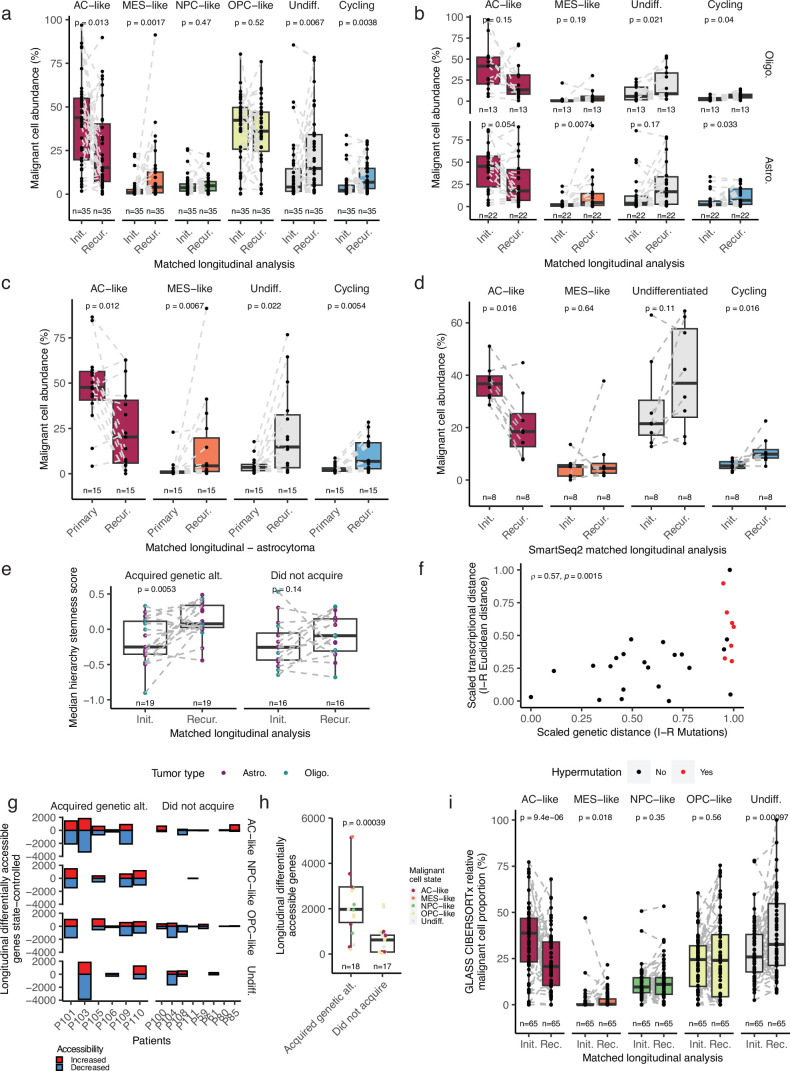
IDH-mutant cellular state evolution is driven by genetics. (**a**) Malignant state abundance is presented for longitudinally collected samples. For all box plots in this figure, the box spans from the first to third quartiles, median values are indicated by a horizontal line and whiskers show 1.5x the interquartile range, and values from a single patient are connected by a dotted line. The P-values presented in panels a-e and i represent two-sided Wilcoxon signed-rank tests. (**b-d**) Longitudinal cell state abundance is shown separated by tumor type (**b**), astrocytoma samples where the initial sample is a primary (i.e., collected from the patient’s first surgery) (**c**), and astrocytoma samples that were also profiled by the plate-based Smart-seq2 single nucleus transcriptomics platform (**d**). (**e**) Median IDH-mutant hierarchy score values are presented per sample stratified by whether there was a longitudinal acquisition of a recurrence-associated genetic alteration. (**f**) Scatterplot represents the scaled genetic distance (bulk DNA) versus scaled pseudobulk (snRNA) transcriptional distance. Two-sided Spearman correlation coefficient and P-value are shown. Color indicates whether sample acquired hypermutation at recurrence. (**g**) The number of state-controlled differentially accessible genes (snATAC) between matched initial and recurrent samples are shown with increased accessibility at recurrence shown in red as a positive number and number of genes with decreased accessibility at recurrence shown in blue (two-sided Wilcoxon rank-sum test, FDR < 0.05, abs(log2FC) > 1). Patients without sufficient nuclei (minimum 100 nuclei) at both time points per cell state are indicated by whitespace. Patients are stratified by those with and without acquired recurrence assoc. genetic alterations. (**h**) The total number of longitudinal differentially accessible genes across cell states are shown for patients with and without acquired genetic alterations. Two-sided Wilcoxon rank-sum test is shown. (**i**) CIBERSORTx estimated malignant cell state abundance for IDH-mutant longitudinal samples profiled by bulk RNA sequencing in the GLASS consortium.

To evaluate how cellular states change over time and following treatment exposure, we compared the malignant-cell-state abundance across time points. We observed significant increases in the MES-like, Undifferentiated and cycling states, which was accompanied by an AC-like decrease, which mirrored differences in grade (Extended Data Fig. 8a). The reduction in AC-like abundance and the increase in the Undifferentiated population were robust and potentially coupled. That is, they were observed in the same subset of samples (Fig. 3c), occurred in both tumour types (Extended Data Fig. 8b) and were found when restricting analysis to astrocytomas at primary diagnosis (Extended Data Fig. 8c) and when assessing malignant-cell states by the Smart-seq2 platform (Extended Data Fig. 8d).

The longitudinal shift towards reduced differentiation and increased cycling was driven by samples with ‘acquired recurrence-enriched genetic alterations’, which is defined as hypermutation, somatic copy number burden increases, small deletion increases or cell cycle alterations (Fig. 3c,d). Indeed, the samples with these acquired genetic alterations demonstrated a significant increase in an independent stemness score, whereas those lacking acquired genetic alterations did not^10^ (Wilcoxon signed-rank test, *P* = 5.3 × 10^–3^; Extended Data Fig. 8e). The MES-like state was an exception, as it increased in matched recurrent samples independent of acquired genetic alterations (Fig. 3d). In further support of the idea that genetic changes drive transcriptional differences, longitudinal mutational genetic distance was positively correlated with malignant-state-controlled pseudobulk transcriptional distance between time points (Spearman’s *ρ* = 0.57, *P* = 1.5 × 10^–3^; Extended Data Fig. 8f). Gliomas with acquired genetic alterations were also more likely to have cell-state-controlled differentially accessible chromatin peaks, which implicated the presence of genome and epigenome co-evolution (Wilcoxon rank-sum test, *P* = 3.9 × 10^–4^; Extended Data Fig. 8g,h). Finally, we verified that acquired genetic alterations were associated with significantly reduced differentiation and increased proliferation by analysing samples from the GLASS IDH-mutant glioma cohort with inferred malignant-state abundance from bulk RNA data (Fig. 3e–g and Extended Data Fig. 8i). Overall, these findings indicate that IDH-mutant gliomas undergo substantial cell-state evolution that is associated with the acquisition of genetic alterations following treatment or at recurrence.

## Genetics and intertumour state variation

**Fig. 4:**
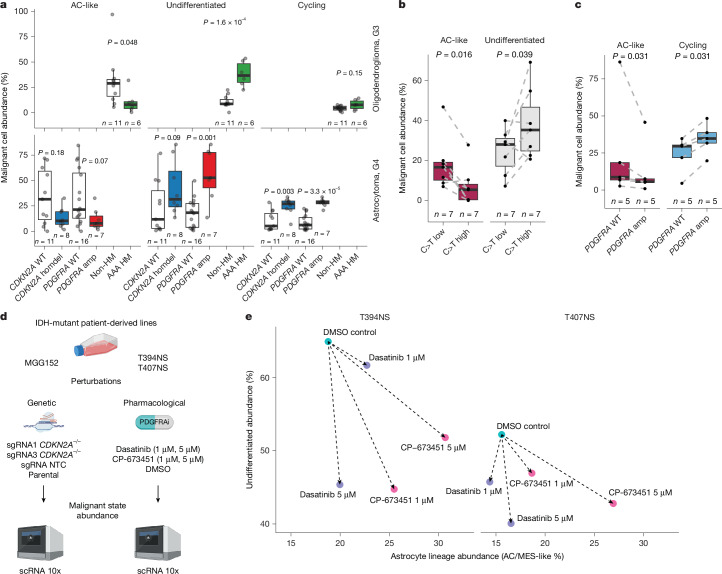
Intertumoural and intratumoural genetic heterogeneity shifts state abundance. **a**, Malignant cell abundance for samples with and without a treatment-associated DNA hypermutation (HM; *n* = 17), *CDKN2A* homozygous deletion (homdel; *n* = 19) or *PDGFRA* amplification (amp; *n* = 23) matched for tumour type and grade. For all box plots in this figure, the box spans from the first to third quartiles, the median values are indicated by a horizontal line and whiskers show 1.5× the interquartile range. Two-sided Wilcoxon rank-sum test associated *P* values are shown for each comparison. AAA, alkylating agent-associated; WT, wild type. **b**, Box plots and individual points for intratumoural hypermutant samples with available 10x Multiome data (RNA + ATAC), whereby high versus low C>T burden nuclei are compared. *P* values reflect one-sided Wilcoxon signed-rank tests based on the directionality of cell-state shifts identified in **a**. Dotted lines connect nuclei from the same sample. **c**, Box plots and individual points for *PDGFRA* amplified (+) and PDGFRA non-amplified (–) nuclei in the same sample. *P* values reflect one-sided Wilcoxon signed-rank tests based on the directionality of cell-state shifts identified in **a**. **d**, Experimental design for IDH-mutant patient-derived cell lines with perturbations for *CDKN2A* loss (genetic; Extended Data Fig. 9h,i) or targeting PDGFRA in *PDGFRA* amplified cell lines (pharmacological; **e**) followed by scRNA-seq. **e**, Undifferentiated-state abundance is plotted against astrocyte lineage (combined AC-like and MES-like states) abundance to highlight a shift towards differentiated states after perturbation. Points reflect malignant-cell-state abundance per experimental condition and colours reflect the pharmacological experimental group. Arrow direction represents the abundance shift from the reference control. Schematic in **d** created in BioRender; Johnson, K. https://biorender.com/bpoemfh (2026).

**Extended Data Fig. 9:**
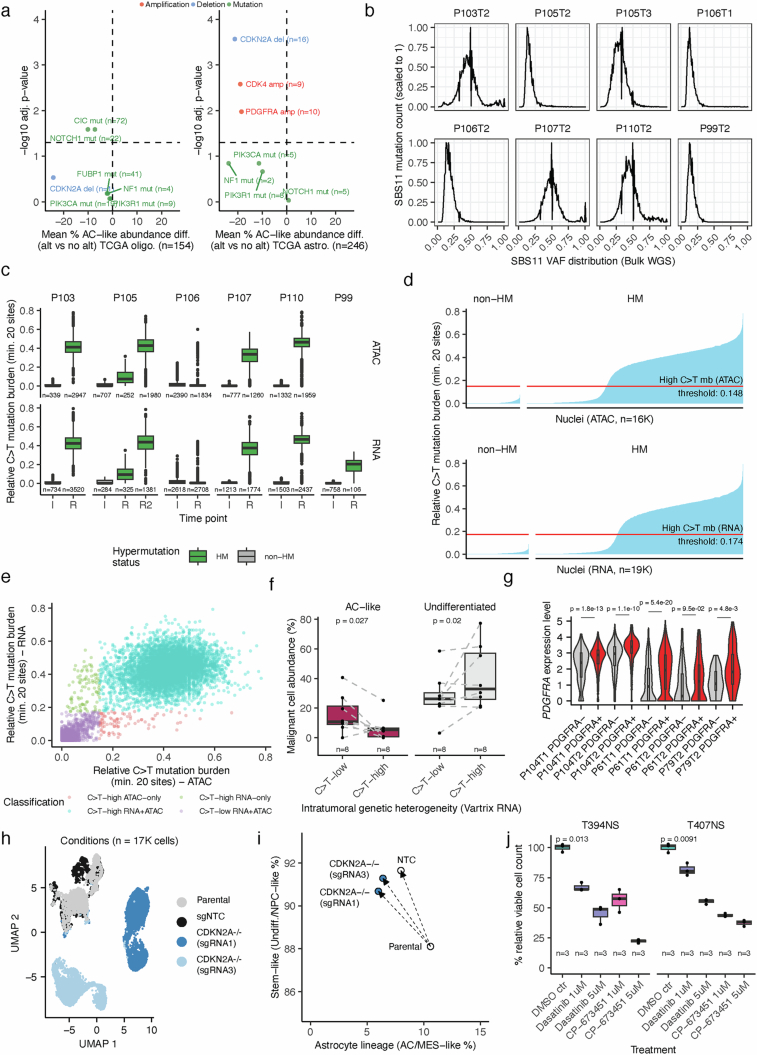
Genetic heterogeneity shapes cellular states. (**a**) TCGA associations between estimated AC-like abundance and gene alterations. Points reflect genetic alterations with the number of samples with alteration shown in parentheses. P-values reflect two-sided Wilcoxon rank-sum test. (**b**) The variant allele frequency (VAF) distribution of SBS11 mutations is presented for each bulk DNA sample with evidence of hypermutation (HM) and sufficient single nucleus data for subclonal analysis. (**c**) The per nuclei relative C>T mutation burden for nuclei colored by whether the bulk samples had evidence of hypermutation. Patient P106 has evidence of treatment-associated hypermutation at both studied time points. For all box plots in this figure, the box spans from the first to third quartiles, median values are indicated by a horizontal line and whiskers show 1.5x the interquartile range. Outlier points are shown. (**d**) Histogram indicates relative C>T mutation burden in non-HM initial samples (left) and HM samples for variants detected in snATAC (top) and snRNA data (bottom). Red line indicates threshold for defining high C>T burden. (**e**) Scatterplot where the relative C>T mutation burden was calculated for both snATAC and snRNA for the same nuclei (n = 9,466) with classification used for each platform. (**f**) Cell state abundance in C>T-low and C>T-high nuclei from the same HM sample in snRNA data, which are connected by dotted lines. P-values reflect one-sided Wilcoxon signed-rank tests. (**g**) *PDGFRA* gene expression violin and box plots are presented for *PDGFRA*- (not amplified) and *PDGFRA* + (amplified) populations. P-values report two-sided Wilcoxon rank-sum tests. (**h**) UMAP plot across experimental conditions targeting CDKN2A in MGG152 cells. sgRNA NTC = non-targeting control. (**i**) Points reflect state abundance per experimental condition and colors reflect experimental group (white = control, blue = CDKN2A−/−). Arrows represent abundance shift from control. (**j**) Percent viability is shown at day 6 of PDGFRA inhibition. P-values from Kruskal-Wallis tests are shown.

We next assessed whether malignant-cell-state abundance across samples was similarly associated with genetic alterations. *CDKN2A* homozygous deletions, *PDGFRA* amplifications and treatment-associated hypermutation have been associated with worse clinical outcomes^34,35^. These genetic drivers of advanced disease were associated with intertumoural cell-state differences (Fig. 4a). For example, grade 4 astrocytomas with *CDKN2A* homozygous deletions or *PDGFRA* amplification had elevated cycling and Undifferentiated populations, respectively. Similarly, grade 3 oligodendrogliomas with treatment-associated DNA hypermutation had expanded Undifferentiated and reduced AC-like populations. We confirmed the non-treatment-related intertumoural associations with genetic alterations in the TCGA cohort (Extended Data Fig. 9a).

## Genetics and intratumour state variation

Having linked genetic alterations to longitudinal and intertumoural cell-state differences, we next asked whether subclonal genetic intratumoural heterogeneity, specifically DNA hypermutation and *PDGFRA* amplification, also shapes cell-state differences within individual samples. To identify nuclei in hypermutant samples with greater mutation burden, we leveraged matched whole-genome sequencing (WGS) data to genotype mutations in the snRNA and snATAC data for a patient’s pre-hypermutant and post-hypermutant samples (Extended Data Fig. 9b,c). As treatment-associated DNA hypermutation events are characterized by large increases in C>T mutations^11,15,35^, we determined a per nucleus relative C>T mutation burden (Extended Data Fig. 9d). To classify nuclei in a hypermutant sample as low or high C>T burden, a background level was determined across all initial samples and established a threshold two times greater than the maximum C>T burden value of the initial samples. Notably, we found consistent high C>T compared with low C>T burden classification from simultaneous snRNA and DNA-based snATAC data (Extended Data Fig. 9e). This analysis demonstrated that high C>T burden nuclei were significantly less differentiated in a sample, a result that supports our intertumoural observations (Fig. 4b and Extended Data Fig. 9f).

Similarly, we examined *PDGFRA* amplifications, intratumoural copy numbers of which have been shown to vary widely^8^. We used Copy-scAT to identify focal *PDGFRA* amplifications in snATAC data and confirmed that these malignant nuclei had higher *PDGFRA* gene expression than in *PDGFRA* wild-type nuclei^36^ (Extended Data Fig. 9g). We observed that *PDGFRA* amplification nuclei (*PDGFRA* amp) had reduced AC-like and increased cycling abundance. This result provides further support for a link between genetic alterations and reduced differentiation (Fig. 4c).

To functionally support the observation that genetic alterations drive malignant-cell-state abundance, we profiled patient-derived IDH-mutant glioma sphere-forming cells (GSCs) by scRNA-seq following genetic and pharmacological perturbations (Fig. 4d). We first assessed whether IDH-mutant cells with CRISPR targeting of p16 (*CDKN2A*^–/–^)^37^ affected cell-state abundance. There was a modest shift towards reduced astrocyte lineage differentiation compared with the parental line and to a lesser extent the non-targeting control line (Extended Data Fig. 9h,i and Supplementary Fig. 4). Next, GSCs derived from two astrocytomas in this cohort that each have *PDGFRA* amplifications^38^ were treated with inhibitors targeting the PDGFRA pathway, including dasatinib (a broad SRC, ABL and PDGFR inhibitor) and CP-673451 (a selective PDGFRα and PDGFRβ inhibitor). Both compounds reduced viable cell counts and induced a shift away from the Undifferentiated population towards increased astrocyte lineage (that is, differentiated) cell-state abundance (Fig. 4e and Extended Data Fig. 9j). Together, these results support the idea that prognostically relevant genetic alterations that are acquired in advanced disease reduce differentiation and expand stem-like states.

## Immune interactions shape MES-like state

**Extended Data Fig. 10:**
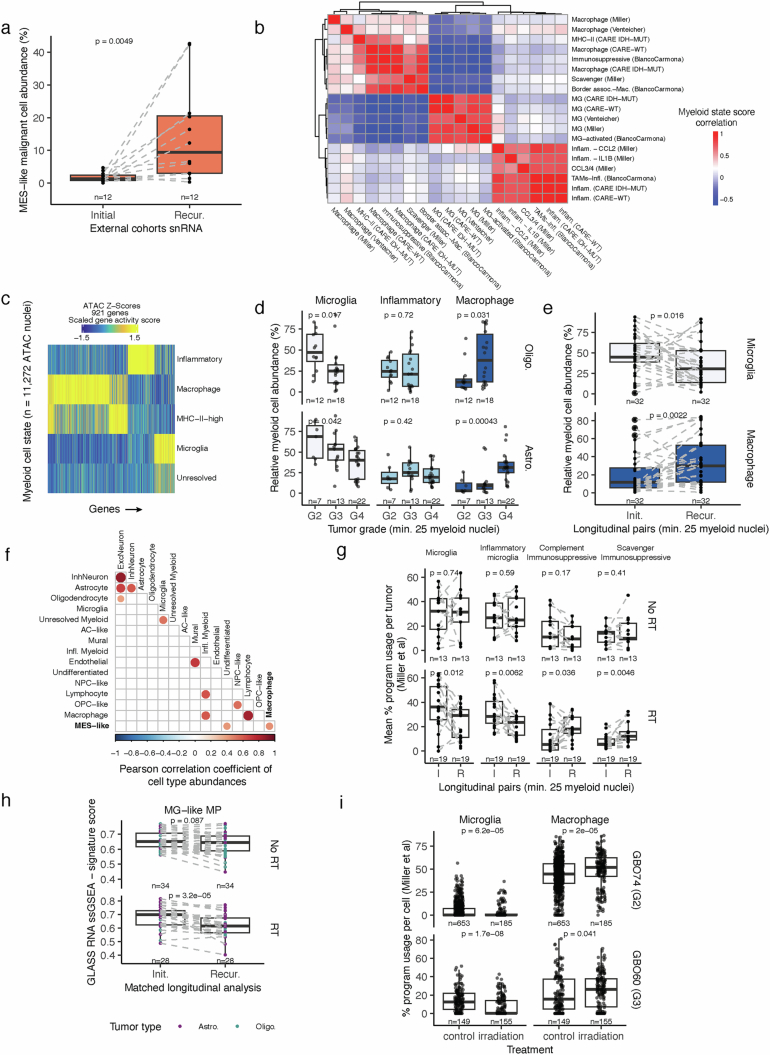
Characterization of myeloid tumor microenvironment. (**a**) Longitudinal MES-like increase in astrocytomas from independent snRNA studies^24,25,31,62^. For all box plots in this figure, the box spans from the first to third quartiles, median values are indicated by a horizontal line and whiskers show 1.5x the interquartile range. Dotted lines connect patient samples. P-value indicates two-sided Wilcoxon signed-rank test in this panel and all other longitudinal analyses in this figure. (**b**) Clustered heatmap based on cell state score correlations between myeloid gene expression metaprograms derived in this study (e.g., Inflammatory, CARE-IDH-MUT) and those from prior glioma myeloid publications^4,23,25,39^. (**c**) Gene accessibility score (snATAC) heatmap for myeloid state marker genes (two-sided Wilcoxon rank-sum test, FDR < 0.05, log2FC >= 0.5). (**d**) The relative myeloid cell state abundance separated by tumor type and tumor grade. The P-values reflect two-sided Wilcoxon rank-sum tests (oligodendrogliomas) and Kruskal-Wallis tests (astrocytomas). (**e**) Longitudinal relative myeloid state abundance differences for microglia and macrophages across patients. (**f**) Correlation heatmap shows pairwise correlation between cell state abundance. Dots indicate associations that were statistically significant (FDR < 0.05), and color indicates correlation strength. (**g**) Mean percent usage for select myeloid programs defined in Miller et al.^39^. Longitudinal analyses are presented for patients are stratified by whether patients received radiotherapy between time points. (**h**) GLASS IDH-mutant samples bulk RNA single-sample Gene Set Enrichment Analysis scores for microglia metaprogram separated based on whether the patient received radiotherapy between time points. (**i**) Selected myeloid program usage scores^39^ in myeloid cells from two IDH-mutant patient-derived organoids (GBOs) that either received irradiation (10 Gy) or control. Tumor grade is indicated in parentheses. Dots represent individual cells. P-values represent two-sided Wilcoxon rank-sum test.

MES-like abundance was significantly increased at recurrence both in our cohort (Wilcoxon signed-rank test, *P* = 0.002; Extended Data Fig. 8a) and in a validation longitudinal astrocytoma snRNA cohort^24,25,31^ (Wilcoxon signed-rank test, *P* = 0.005; Extended Data Fig. 10a). However, the MES-like cellular state shift was not associated with acquired genetic alterations, which suggests that extrinsic factors, including the tumour microenvironment (TME), influence shifts. To better understand TME cellular diversity, we applied a NMF-based approach to define non-malignant gene expression metaprograms^18^. Owing to the relatively high abundance of myeloid cells, an established link with the IDH wild-type MES-like state and their recognized intratumoural heterogeneity^4,14,25,30,39^, we focused our subsequent TME analyses on the myeloid compartment. We assigned each myeloid nuclei to a specific state on the basis of relative expression of the identified metaprograms^18,20,23^ (Supplementary Table 6).

**Fig. 5:**
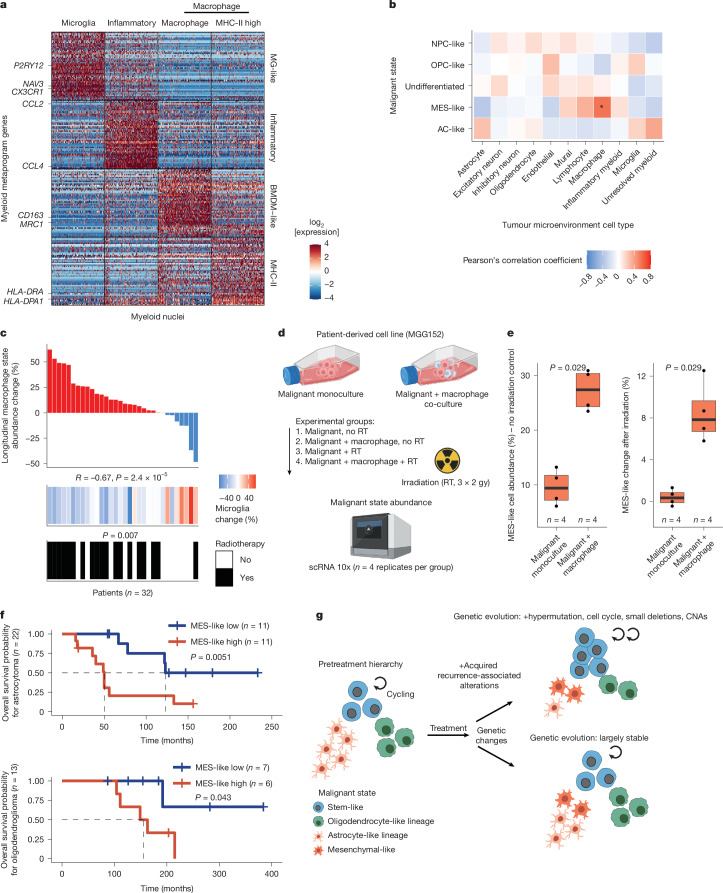
Inflammatory microenvironments and injury response at recurrence. **a**, Gene expression heatmap for nuclei assigned to a myeloid cell state (*x* axis) based on the identified myeloid NMF metaprograms (*y* axis). Selected genes are presented per metaprogram. **b**, Heatmap of two-sided Pearson’s correlation coefficients depict the relative change (recurrence – initial) in tumour microenvironment abundance (*x* axis) and the relative change in malignant-state abundance. The asterisk indicates Pearson’s correlation adjusted *P* = 0.03. **c**, Longitudinal change in relative macrophage abundance per a waterfall plot, with each patient represented by an individual bar. Colour indicates whether the matched longitudinal change was an increase (red) or decrease (blue) at recurrence. The first row reflects the relative longitudinal change in microglia and the second row reflects whether the patient received radiotherapy (RT) between the two time points. *P* values reflect the Pearson’s correlation (*R* = −0.70), longitudinal differences based on radiotherapy treatment (two-sided Wilcoxon rank-sum test). **d**, Experimental design for IDH-mutant patient-derived cell lines cultured in either malignant monoculture conditions or co-cultured with mouse macrophages that either received or did not receive 3 days of 2 Gy irradiation. Conditions were repeated across four biological replicates and subjected to scRNA-seq. **e**, MES-like malignant-state abundance across monoculture versus co-culture conditions under no irradiation control conditions (left). The change in MES-like abundance following irradiation across monoculture and co-culture conditions (right). *P* values represent two-sided Wilcoxon rank-sum tests. **f**, Kaplan–Meier plot for overall survival for median levels of MES-like-state abundance at recurrence across both astrocytoma (top) and oligodendroglioma (bottom) samples. *P* values indicate log-rank tests. **g**, The longitudinal IDH-mutant malignant state hierarchy model shifts towards reduced differentiation in samples that acquired genetic alterations versus a more stable-cell-state composition in samples without these alterations. Independently, samples have a shift towards a more macrophage-rich myeloid compartment and an increase in the reactive MES-like state. Schematic in** d** created in BioRender; Johnson, K. https://biorender.com/ppiph3l (2026). Schematic in **g** adapted from ref. ^8^, Springer Nature America.

The myeloid subpopulations identified were brain-resident microglia (MG-like; *CX3CR1*, *P2RY12* and *NAV3*), inflammatory myeloid (*CCL3*, *CCL4* and* CD83*), bone-marrow-derived macrophages (BMDM-like; *CD163* and* MRC1*), a subset defined by high major histocompatibility class II expression (MHC-II; *HLA-DRA* and *HLA-DPA1*) or nuclei that could not be confidently assigned to a subpopulation (unresolved myeloid, Fig. 5a). Myeloid-state annotations were supported by positive correlations with previously reported myeloid signatures across IDH-mutant and IDH wild-type samples^4,23,25,39^ (Extended Data Fig. 10b). Bone-marrow-derived macrophages and the MHC-II-high myeloid population were collapsed into a single macrophage state owing to their similar gene expression and chromatin accessibility profiles (Fig. 5a and Extended Data Fig. 10c). Higher-grade samples showed increased macrophage abundance and reduced microglia abundance relative to lower-grade samples, a pattern that was also observed at recurrence (Extended Data Fig. 10d,e).

To better understand the microenvironmental factors that shape malignant cell abundance, we examined the associations between malignant and TME cell-state abundances, including the myeloid states. As previously observed^30^ in IDH wild-type glioblastomas, the MES-like state and macrophage abundances were significantly correlated, but no significant positive associations for other malignant and TME states were observed (Extended Data Fig. 10f). This association was also seen longitudinally, as increased macrophage abundance was significantly correlated with increased MES-like abundance (*R* = 0.56, adjusted *P* = 0.03; Fig. 5b). To further dissect the longitudinal myeloid shift, we separated patients on the basis of clinical factors such as treatment status. Myeloid subpopulations shifted towards a decrease in microglia and an increase in macrophage abundance, particularly with radiotherapy treatment (Wilcoxon rank-sum test, *P* = 0.007; Fig. 5c). To identify clinical factors most strongly associated with this state shift, we performed multiple linear regression, which confirmed that treatment with radiotherapy was significantly associated with microglia decreases and macrophage increases while adjusting for grade increase, surgical interval, alkylating agent treatment and tumour type (Supplementary Table 7). Application of independent myeloid cell signatures from a glioma myeloid cell atlas^39^ resulted in consistent longitudinal changes in samples treated with radiotherapy (Extended Data Fig. 10g). An analysis of the GLASS bulk RNA dataset further supported longitudinal reduction in microglia expression specifically following radiotherapy treatment (*P* = 3.2 × 10^–5^, Wilcoxon signed-rank test, Extended Data Fig. 10h). To validate these observations, we performed parallel monoculture and co-culture experiments between macrophages and an IDH-mutant GSC (MGG152), each conducted with and without irradiation, followed by scRNA-seq. We observed significant increases in MES-like abundance in co-culture with macrophages versus monoculture (Wilcoxon rank-sum test, *P* = 0.028, Fig. 5d, left). Moreover, a MES-like abundance increase following irradiation was significantly higher in the co-culture condition (Wilcoxon rank-sum test, *P* = 0.028; Fig. 5d, right). We also performed irradiation experiments using patient-derived IDH-mutant organoids followed by scRNA-seq and observed expression patterns consistent with myeloid cells in the organoids taking on a more macrophage-like state in the irradiation condition (Extended Data Fig. 10i). Together, these data indicate that although effective at improving patient outcomes^40^, radiotherapy facilitates changes in the myeloid compartment that may influence malignant states and treatment response.

Collectively, our results suggest that genetics may drive malignant cells towards less differentiated and more proliferative states, whereas the increased MES-like abundance coincides with inflammatory microenvironments. Last, we assessed whether malignant-state abundance could provide additional information regarding clinical outcomes. We observed a significant association between a high MES-like fraction in the matched recurrent sample and reduced overall survival in both astrocytoma and oligodendroglioma (Fig. 5f). This result remained significant when controlling for other prognostic factors such as age at diagnosis, tumour type and tumour grade (Supplementary Table 8a,b). In summary, the longitudinal malignant-state shifts highlight that IDH-mutant glioma progression is fuelled by parallel changes in genetics and the microenvironment that result in more aggressive recurrent disease (Fig. 5g).

## Discussion

We reconstructed a multilayered evolution of IDH-mutant gliomas to better understand disease progression and treatment resistance. Through multiomic single-nucleus profiles collected across multiple time points, we identified an expanded catalogue of IDH-mutant glioma malignant-cell states that are epigenetically encoded and shift towards increased proliferation and reduced differentiation with disease progression. Integrative analyses with DNA-seq further revealed that the acquired recurrence-associated genetic alterations, including treatment-associated events (for example, alkylating agent-associated hypermutation), drive the transcriptional differences that contribute to therapeutic resistance. Beyond genetically driven cell-state changes, we observed tumour microenvironment influences on the MES-like malignant-cell state, primarily at recurrence. The increased MES-like abundance and elevated activity of transcription factors involved in stress responses highlight potential non-genetic evolutionary trajectories in IDH-mutant gliomas. These adaptable epigenetic and transcriptional programs probably poise cells to respond to increased inflammatory stimuli and reactive microenvironments that follow conventional therapies^14^. More broadly, the presence of NPC-like, OPC-like, AC-like and MES-like states, a genetic influence on state abundance and a transition towards a more macrophage-rich immune compartment in advanced IDH-mutant disease are patterns reminiscent of IDH wild-type glioblastomas, which raises the possibility of shared cellular lineages and reactive states across adult glioma tumour types^3,41^.

Previous single-cell studies have reported three malignant subpopulations in IDH-mutant glioma, including stem-like, astrocyte-like and oligodendrocyte-like cells, but were limited to single time points or modest sample sizes^4,7,8,10,24,25,33,42,43^. Longitudinal bulk sequencing studies, such as those performed by the GLASS consortium, have shown that acquired genetic alterations coincided with increased proliferation yet lacked the resolution to detect cell-state-specific changes^9,12,14^. Thus, our longitudinal single-nucleus dataset enabled us to address a key question in the field: whether pre-existing malignant states are selected for or whether new states emerge. Our findings provide evidence for both possibilities, as proliferative, stem-like states expanded owing to genetic alterations, whereas the emergence of the MES-like state was decoupled from genetic evolution. Future investigations should determine whether genetic alterations arise preferentially in less differentiated cells that clonally expand or whether they directly drive more stem-like expression.

Our observations that acquired genetic alterations expand stem-like states have implications for current and emerging therapies. Although treatments, including alkylating agents, provide established survival benefits^40,44^, resistance may occur through acquired genetic alterations, including DNA hypermutation. For targeted treatments, early mutant-IDH-inhibitor treatment-response studies observed a depletion of the stem-like population and induction of lineage differentiation, whereas genetic events such as *NOTCH1* mutation were found to modify inhibitor response^24,42^. Together with our observations, these data suggest that genetic and cell-state characterization may identify patients most likely to respond to treatment strategies aimed at depleting proliferative stem-like cells and promoting lineage differentiation.

To support the generalizability of our findings, we validated key observations across hundreds of independent single-cell and bulk IDH-mutant glioma samples. Nevertheless, limitations exist. Our study design required patients to have undergone multiple resections, which skewed the cohort towards younger patients with more favourable outcomes. Moreover, variability in treatment across patients may have influenced treatment-associated findings. Spatial rather than longitudinal heterogeneity may also account for some observations. Finally, the association between the MES-like state and reduced overall survival aligns with previous reports but requires validation in larger cohorts^14,43,45^.

Collectively, these findings advance our understanding of IDH-mutant glioma evolution by demonstrating that disease progression is shaped by interconnected genetic, epigenetic and microenvironmental factors. Future studies, including responses to mutant-IDH inhibitors, may use these findings as a point of reference to further interrogate patterns of IDH-mutant glioma evolution and treatment response towards improving patient outcomes.

## Methods

### Human glioma samples

Frozen glioma tissue specimens with the *IDH1* or *IDH2* mutation were collected with informed consent from the following tissue source sites: Saint Joseph’s Hospital, the Luxembourg Institute of Health-NORLUX (LIH-NORLUX), the MD Anderson Cancer Center (MDACC), Seoul National University (SNU) Hospital and the Pitié-Salpêtrière Hospital. Sample collection was approved by each tissue source site’s Institutional Review Board (IRB). The IRB protocol numbers of respective institutes are as follows; MDACC, 2012-0441; LIH-NORLUX, 201201/06; SNU, H-2004-049-1116; Pitié-Salpêtrière Hospital, 96-900; and Saint Joseph’s Hospital, 2020-NHSR-0084. *IDH* mutation status and chromosome 1p and 19q co-deletion status were obtained from pathology reports and confirmed by DNA-seq in this study when available. Detailed clinical information, including patient sex, age at diagnosis and treatment information, is provided in Supplementary Table 1. Astrocytoma samples with evidence of *CDKN2A* homozygous deletion were assigned grade 4 status in accordance with the 2021 WHO diagnostic criteria^1^. Adjacent fragments from each glioma sample were aliquoted for single-nucleus assays and bulk tissue nucleic acid isolation when sufficient material was available.

### Tissue processing and nucleus isolation

Isolation of nuclei from frozen glioma samples was performed by three separate laboratories using two different protocols. Frozen glioma samples from the same patient were always processed together. Laboratory 1 and laboratory 2 processed the cohorts from MDACC and Pitié-Salpêtrière Hospital using a previously described protocol^46^. In brief, frozen samples were thawed and mechanically dissociated in 0.49% CHAPS with salts and Tris (ST) buffer (10 mM Tris-HCL pH 7.5, 146 mM NaCl, 1 mM CaCl_2_ and 21 mM MgCl_2_). Single-nucleus suspensions were filtered using a 40 μm strainer, centrifuged at 500*g* for 5 min and resuspended in ST buffer with 0.01% BSA (Sigma). Final nucleus suspensions were stained by Trypan blue and counted using a haemocytometer. The number of nuclei was then determined for use in a 10x Genomics workflow (that is, targeted nucleus recovery) or a FACS-sorting for Smart-seq2 workflow.

Laboratory 3 processed the cohorts from LIH-NORLUX, SNU and Saint Joseph’s Hospital with a protocol using EZ lysis buffer (Millipore Sigma). In brief, frozen samples were thawed and mechanically dissociated in Nuclei EZ lysis buffer via dounce homogenization. The solutions were incubated on ice for 5 min and mixed 1–2 times during incubation. Single-nucleus suspensions were filtered through a 70 μm strainer and centrifuged at 500*g* for 5 min at 4 °C, resuspended in Nuclei EZ lysis buffer and incubated on ice for 5 min. The solutions were centrifuged at 500*g* for 5 min at 4 °C and resuspended 3 times in 1% BSA and 0.2 U μl^–1^ RNase inhibitor and PBS buffer. The final suspension of nuclei was stained with DAPI, filtered through a 40 μm strainer and counted using a Countess II automated cell counter (Thermo Fisher Scientific). The number of nuclei was then determined for use in 10x Genomics 3′ RNA or Multiome workflow.

### Bulk DNA-seq

Bulk DNA-seq was performed in a tissue source site-specific manner. For samples from the MD Anderson Cancer Center, DNA was extracted from each frozen glioma sample and blood sample corresponding to the patients using a DNeasy Blood & Tissue kit (Qiagen). The genomic DNA (100–250 ng) was acoustically sheared using an ultrasonicator (Covaris), targeting 150 bp fragments. Library preparation was performed using a KAPA HyperPrep kit (KAPA Biosystems) followed by clean-up using AMPure XP beads (Beckman Coulter). Exome capture was performed using a custom exome bait (manufactured by Twist Biosciences to The Broad Institute’s specification). Captured libraries were sequenced with 150-bp paired-end sequencing on a NovaSeq 6000 (Illumina). For the samples from Pitié-Salpêtrière Hospital, after the DNA was fragmented using a LE220 ultrasonicator (Covaris) and size selected, library preparation and capture were performed using a Twist Human Core Exome kit (Twist Bioscience) according to the manufacturer’s protocol. Sequencing was performed on a NovaSeq 6000 (Illumina). WGS data were generated for frozen samples from Saint Joseph’s Hospital, SNU and LIH-NORLUX. In brief, DNA was extracted from each glioma sample using an AllPrep DNA/RNA Minikit (Qiagen) for samples with sufficient tumour tissue and matched normal blood when it was available. DNA was sheared to 400 bp using a LE220 ultrasonicator (Covaris) and size-selected using AMPure XP beads (Beckman Coulter). Whole-genome libraries were prepared and sequenced with 150-bp paired-end sequencing on a NovaSeq 6000 (Illumina). Whole-exome sequencing was additionally performed for Saint Joseph’s Hospital samples using Agilent SureSelect Human All Exon v7 capture kit, followed by 150-bp paired-end sequencing on a NovaSeq 6000 (Illumina). The WGS data for the SNU cohort was previously reported^14^.

### snRNA-seq and ATAC–seq

A 10x Chromium Single Cell 3′ Reagent kit v.3 (10x Genomics) was used according to the manufacturer’s protocol. In brief, nuclei were loaded on a Chromium chip (10x Genomics) with a target cell recovery of 6,000–8,000 nuclei and processed in a Chromium Controller. Single-nucleus samples were partitioned into gel beads-in-emulsion (GEMs), followed by RNA reverse transcription with barcoding. Libraries were created by breaking GEMs and pooling barcoded fractions, cDNA amplification, fragmentation and attachment of a sample index and adapter and sequenced on a Nextseq500 or Novaseq (Illumina). For three samples in the Saint Joseph’s Hospital cohort, a 10x Chromium Single Cell 3′ Reagent kit v.3 was used. For LIH-NORLUX, SNU and the remainder of the Saint Joseph’s Hospital samples, nuclei were loaded on a Chromium chip with a target cell recovery of 6,000 nuclei for 10x single-cell Multiome ATAC and gene expression according to the manufacturer’s protocol.

### Nucleus sorting for Smart-seq2

Single-nucleus samples stained using Vybrant DyeCycle Ruby stain (Thermo Fisher Scientific) were sorted on a FACS Aria Fusion sorter (Becton Dickinson) with a 640 nm laser (670/14 filter). After doublets were discriminated, singlet nuclei were selected with Ruby positive and were sorted into 96-well plates containing TCL buffer (Qiagen) with 1% β-mercaptoethanol. After sorting, plates were immediately frozen on dry ice and stored at –80 °C before Smart-seq2 experiments.

The Smart-seq2 protocol was performed as previously published with slight modification for single-nucleus profiling^3,47^. In brief, on 96-well plates, RNA derived from single nuclei was first purified with Agencourt RNAClean XP beads (Beckman Coulter). Then, Oligo-dT primed reverse transcription was performed using Maxima H Minus reverse transcriptase (Thermo Fisher Scientific) and locked TSO oligonucleotides (Qiagen). This was followed by PCR amplification (22 cycles) using KAPA HiFi HotStart ReadyMix (KAPA Biosystems) with subsequent Agencourt AMPure XP bead purification. Libraries were tagmented using a Nextera XT Library Prep kit (Illumina) with custom barcode adapters. Pooled libraries were sequenced on a NextSeq 500 or Novaseq 6000 sequencer (Illumina).

### Somatic variant detection and analysis

DNA-seq alignment, fingerprinting, somatic variant detection (Mutect2) and copy number segmentation were performed in accordance with the Genome Analysis Toolkit (GATK) best practices using GATK (v.4.0.10.1), as previously described^11,14^. DNA fingerprint analysis using ‘CrosscheckFingerprints’ (Picard) confirmed that all samples belonging to a patient came from the same individual, which indicated that there were no sample mismatches in this study. Patient glioma samples without matched normal blood were analysed for CNAs, and tumour-only single-nucleotide variant detection was performed with a panel of normal references according to GATK best practices. Tumour-only somatic variants were used only to confirm *IDH1* mutation status and to identify any hypermutation events. To identify samples with increased small deletion mutation burden, the following thresholds were applied: the recurrence-specific small deletion mutation burden needed to be >0.2 mut per Mb sequenced and a >0.1 mut per Mb increase when comparing all small deletion variants in the matched recurrence versus the initial glioma sample. Mutational signature estimation was performed using COSMIC signatures as previously published^11,48^, including the SigProfilerExtractor tool (v.1.2.1). For samples with matched blood, treatment-associated DNA hypermutation was determined by an elevated mutation burden (>10 mut per Mb) and COSMIC SBS11 signal (alkylating agent-associated signature). For samples without matched blood, there were no samples with evidence of hypermutation. Samples with acquired increases in the proportion of the genome with SCNAs were defined as samples with a >50% increase in total SCNA burden at matched recurrence relative to the initial sample. In samples without bulk DNA samples at both time points, acquired SCNA burden increase was determined using the snRNA-inferred CNA profiles. Focal *PDGFRA* amplifications were determined by a combination of GATK CNA detection, DNA methylation array, Sequenza^49^ and AmpliconArchitect, which is part of AmpliconSuite-pipeline (v.0.1344.2), which was run using default settings^50^.

### snRNA-seq analysis

All snRNA-seq 10x data were preprocessed with cellranger count (cellranger v.6.1.2) using the GRCh38-2020-A reference transcriptome downloaded from the 10x website and with ‘include introns’ set to true. Filtered feature count matrices were loaded into R using Seurat^51^ (v.4.1.3) with the Read10X function. To retain high-quality nuclei, nuclei with <1,000 genes detected, >10,000 genes detected or >5% mitochondrial reads were filtered out. DoubletFinder (v.2.0.3) was then used to identify nuclei doublets independently in each sample, and the doublets were subsequently removed^52^. The expression data were then processed by normalization, scaling, PCA based on the 5,000 most highly variable genes, Harmony batch correction with the batch variable set to patient (all samples belonging to a patient were processed in the same 10x run) and laboratory (for astrocytomas), UMAP and clustering using the Louvain algorithm in Seurat with resolution set to 0.6 (ref. ^53^). Cell-type annotation was performed on the basis of gene expression markers for each cluster and confirmed via mapping to a normal reference brain atlas (Azimuth) that provided a per-nuclei cell-type prediction. This process identified clusters with a small number of nuclei expressing multiple cell-type signatures. These nuclei were presumed to be residual doublet populations and were removed. Azimuth integration provided classifications at the cell class level (that is, neuronal versus non-neuronal) and a more granular subclass (astrocyte, oligodendrocyte precursor cell, endothelial, among others) that confirmed appropriate assignment by expression clustering.

### Malignant and non-malignant cell-type assignment

Malignant status was confirmed by integrating expression-based clusters with snRNA-inferred copy number. Specifically, large-scale CNAs were determined for each sample separately using the software inferCNV of the Trinity CTAT Project (https://github.com/broadinstitute/inferCNV), with the reference nuclei set to myeloid and oligodendrocytes from that sample. For one sample without sufficient myeloid or oligodendrocyte nuclei, its matched initial sample’s reference nuclei were used to infer copy number. Predicted copy number altered regions based on a moving average of a 101 gene window were determined with the hidden Markov model (HMM) parameter enabled. The HMM-predicted CNA levels were extracted to define three metrics used in defining malignant and non-malignant nuclei:

1. CNA signal per chromosome: the proportion of genes gained and lost per chromosome that produces two chromosome-specific metrics (for example, the proportion of chromosome 1 with copy gain and the proportion of chromosome 1 with copy loss) that reflected the CNA burden per chromosome.
2. CNA signal per nucleus: the mean of all per-nucleus chromosome CNA signals, which reflects the total CNA burden of that nucleus independent of gains or losses.
3. CNA correlation: the Pearson’s correlation between the CNA signal per chromosome values and the average per chromosome CNA profile of all expression-based malignant nuclei or bulk DNA CNA profile from the same glioma sample.

To assign an integrated expression and to inferCNV malignant cell definition, different strategies were used for oligodendrogliomas and astrocytomas, respectively. For oligodendrogliomas, the presence or absence of the tumour-type-defining chromosome 1p and 19q co-deletion was used to confirm malignant status. Specifically, nuclei that were annotated as malignant by gene expression clusters that had >0.15 proportion of chromosome 1 or chromosome 19 with copy number loss were retained as malignant, whereas nuclei not meeting this criterion were classified as ‘unresolved’. For the nuclei annotated as non-malignant by expression clustering, that were not assigned to the neuronal class by Azimuth integration and had >0.15 proportion of chromosome 1 and chromosome 19 with copy number loss (that is, a clonal CNA event) were classified as unresolved. For astrocytomas, nuclei annotated as malignant by gene expression that had a >0.3 CNA correlation coefficient or a per nucleus CNA signal of >0.15 were retained as malignant, whereas nuclei not meeting this criterion were classified as unresolved. For the nuclei annotated as non-malignant by expression clustering, that were not assigned to the neuronal class by Azimuth integration and had >0.3 CNA correlation or >0.15 per nucleus CNA signal were classified as unresolved. Nuclei classified as unresolved were excluded from downstream analyses. Furthermore, for samples in which bulk DNA CNA data that passed quality control or had purity metrics were available, a per-chromosome metric representing the fraction of the chromosome gained or lost in bulk DNA was determined and correlated with the per-chromosome CNA signal (inferCNV) of each nucleus, which provided an additional layer of assessment.

### Deriving metaprograms from gene expression data

To capture the heterogeneity among nuclei of the same type, we used NMF^54^, a method previously applied to identify variability in single-cell and single-nucleus expression data^18–20^. NMF was performed on the relative expression values of each sample independently after setting negative values to zero. The NMF algorithm requires the pre-definition of the *k* parameter, which represents the expected number of latent features in the data. As *k* varies between samples and is largely unknown, we applied the NMF algorithm to each sample using a range of values (3–10). Each of these NMF programs was summarized by the top 50 genes based on NMF coefficients. The process of deriving metaprograms from the NMF programs has been previously detailed^18^ and is described in brief here. The method first eliminates NMF programs that are either not robust (that is, they do not recur in or across samples) or redundant in a sample (that is, they significantly overlap with other NMF programs in the same sample). The robust NMF programs are then clustered based on Jaccard similarity using a clustering algorithm that iteratively assesses the degree of overlap between programs, combining highly overlapping ones into a cluster. Each cluster is defined by the top 50 most recurrent genes to form a metaprogram.

The algorithm identified 12 malignant metaprograms, which were annotated using functional enrichment analysis (for example, GO terms, mSigDB Hallmark gene sets and gene sets derived from normal brain development datasets). Metaprograms were excluded if they reflected quality control issues (for example, mitochondrial or ribosomal genes). Metaprograms were also excluded that were not detected in both tumour types with a minimum 25% contribution from at least one tumour type. The metaprograms derived from the primary analysis included OPC, AC1, AC2, MES and CC. We repeated the metaprogram analysis for the Undifferentiated population and identified a NPC metaprogram, which was added to the malignant metaprograms used for cell-state assignment.

To provide an orthogonal description of the gene-based metaprograms, we modified the NMF-based algorithm by considering single-nucleus pathway activities instead of gene expression. To do this, we identified 6,086 gene sets collected from Hallmark, Gene Ontology and KEGG that had a minimum of 15 genes that also overlapped with the snRNA data. Pathways were scored independently for each malignant nuclei in a sample. The NMF-based approach to determine metaprograms was then applied as defined above for the pathway activities scores. This resulted in 11 pathway-based metaprograms (PMPs) that were named according to the most represented pathway activities, which included pathways such as mitochondrial energy production as well as glycolysis and stress PMPs shown in Extended Data Fig. 5i.

### Cell-state scoring and assignment of nuclei to states

The Seurat function AddModuleScore was used to score each 50-gene metaprogram against a background set of genes to derive a score. Malignant nuclei were scored for the NMF metaprograms independently for each sample. To facilitate cell-state classification, we generated 20 shuffled expression matrices by sampling each time 5,000 nuclei and shuffling the expression values for each gene. We then scored each shuffled matrix for the NMF metaprograms, thereby producing 100,000 normally distributed scores for each expression program. These served as null distributions for cell-state classification. For each nucleus, we computed a *P* value for each of the expression programs with a* Z*-test (R’s pnorm function) using the statistics of the null distributions that we previously generated. We adjusted all *P* values for multiple testing using the Holm method. Each cell was classified into a specific state if the adjusted *P* value computed for that state was <0.05. Nuclei that achieved an adjusted *P* < 0.05 for multiple states were assigned to the state for which they scored maximally. Nuclei that did not achieve an adjusted *P* < 0.05 for any of the states were assigned an Undifferentiated state. Unlike the other metaprograms that are positively defined by metaprogram expression, the Undifferentiated state is defined by the absence of high AC-like, MES-like, NPC-like and OPC-like expression. Although we observed that Undifferentiated nucleus transcriptional profiles are consistent with an intermediate or transitional stem-like state, it is possible that rare states not commonly found across patients with an *IDH* mutation are assigned to the Undifferentiated state. Nuclei were assigned a ‘cycling’ state on top of their cellular state if they achieved an adjusted *P* < 0.05 for the cell cycle metaprogram and non-cycling otherwise. Cell-state assignment for the external scRNA-seq, internal Smart-seq2 cohorts, and myeloid nuclei used a threshold of a nominal *P* < 0.05. Separately, malignant nuclei were scored for previously published malignant and non-malignant signatures using Seurat’s AddModuleScore function. Following gene set score calculation, the IDH-mutant hierarchy coordinates for stemness and lineage scores were calculated as previously described^10,24^. In brief, the lineage scores were determined by the maximum AC-like and OC-like gene set program, whereas the stemness score reflected the difference in the stemness gene set and lineage score.

### Pseudobulk snRNA analyses

Pseudobulk transcriptional profiles for PCA and differential expression from snRNA data were generated with the R package presto’s collapse_counts function (https://github.com/immunogenomics/presto). Pseudobulk profiles were constructed for malignant state-specific profiles with a minimum number of 20 nuclei per sample per state. Differential expression for pseudobulk profiles was performed using presto’s pseudobulk_deseq2 function with a design matrix that included patient, tumour type and time point. Enrichment of differentially expressed genes was performed using the R package fgsea (https://github.com/ctlab/fgsea/) and gene sets from MsigDB’s Hallmark gene sets. The pseudobulk profiles were log_2_-normalized, and the top 1,000 most variable genes were used as input for PCA.

### Analysis of external IDH-mutant scRNA-seq and snRNA-seq datasets

Re-analysis of external cohorts were separately preprocessed from raw counts. When unavailable, raw counts were recovered from normalized counts using a script from GitHub (https://github.com/immunitastx/recover-counts). Cell-type identification was either provided with the study or was determined via dimensionality reduction and clustering followed by copy number inference to confirm malignant status. Derivation of gene expression metaprograms for malignant cells or nuclei and malignant-cell-state classification was performed as described above for the CARE IDH-mutant cohort. The gene expression metaprograms for CARE IDH wild-type glioblastoma were determined by the same laboratories for 121 IDH wild-type glioblastomas using the same profiling and analytical methods^23,55^. The Jaccard similarity index was used to assess overlap of specific genes used across the metaprograms derived from different cohorts.

### Bulk RNA deconvolution and cell cycle scoring

Cell-type abundances were estimated from bulk RNA-seq gene expression matrices using CIBERSORTx^14,56^. In brief, a reference signature matrix was created from the CARE IDH-mutant snRNA dataset for samples with accompanying bulk RNA-seq using the ‘Create Signature Matrix’ module on the CIBERSORTx webserver (https://cibersortx.stanford.edu) with default parameters, except the minimum expression was set to 0.5 per instructions for 10x data. We downsampled our larger single-nucleus gene expression matrix and used the major cell types and the malignant-cell states to construct the signature matrix. Transcriptionally similar cell types (for example, excitatory and inhibitory neurons, mural and endothelial cells) were collapsed following initial assessment of CIBERSORTx deconvolution performance. Cell-type abundances were then imputed using the ‘Impute Cell Fractions’ module in single-cell mode (S-mode batch correction) and 100 permutation parameters for TCGA and GLASS bulk RNA expression datasets^13,14^. Cell-type abundance inference performance in bulk RNA samples was assessed using Pearson’s correlation coefficient for glioma samples for which both snRNA and bulk RNA-seq were collected from the same tumour sample. Malignant cell cycle and myeloid metaprograms in bulk RNA-seq were assessed using a ssGSEA score from the R package GSVA^57^.

### snATAC–seq analysis

snATAC–seq data were available for a subset of the CARE IDH-mutant cohort from 10x Multiome data. snATAC–seq data were processed using Cell Ranger ARC (v.2.0.2). The resulting fragment files were imported by ArchR^58^ and Arrow files were created with minimum quality metrics of transcriptional start site (TSS) enrichment of 4 and 1,000 fragments. The ArchR function addDoubletScores with *k* = 10, and filterDoublets was used to identify and remove doublets. Following doublet removal, nuclei were filtered down those that passed quality control for both snRNA and snATAC. The addIterativeLSI function was used to compute an iterative latent semantic indexing dimensionality reduction for a tile matrix of both all nuclei and then separately restricted to only malignant nuclei. For malignant nuclei, Harmony batch correction was performed by sample. During preprocessing, ArchR created a gene activity score, which reflects the regulatory region chromatin accessibility around a gene. For differential accessibility analyses, cell-state-specific comparisons of both gene activity scores and peak matrices were performed using a Wilcoxon rank-sum test adjusted for bias in TSS enrichment and the log_10_[number of fragments], as implemented by ArchR. TF activity was predicted on a per-cell basis using chromVAR to calculate a *z*-score based on the per-cell accessibility of a given TF motif (cisbp) that deviates from the expected accessibility based on the average of all nuclei. The R package Copy-scAT was used to determine focal *PDGFRA* CNA in the snATAC data^36^. This analysis was restricted to samples with a focal *PDGFRA* amplification determined by WGS and, in one case for which the whole genome was unavailable, DNA methylation array. In brief, the Copy-scAT function ‘identifyDoubleMinutes’ was used to detect focal copy number amplifications per malignant nucleus from a read depth normalized and scaled fragment matrix. This method uses a mean-variance changepoint analysis to detect amplified signal across each chromosome and amplified regions containing the *PDGFRA* locus were extracted for downstream analysis.

### Single-nucleus genotyping of bulk WGS-derived mutation calls in hypermutant samples

To detect bulk DNA-seq variants in the single-nucleus data, VarTrix was used (v.1.1.22) to extract single-nucleus variant information (https://github.com/10XGenomics/vartrix). VarTrix takes an input variant call format (VCF) file, single nucleus bam file, fasta file and outputs whether the variant had no call (no reads detected), ref/ref (only reference allele detected), alt/alt (only alternative allele detected) or alt/ref (both alleles detected) in a single cell. VarTrix was run both on snRNA and snATAC data and only applied to patients who had at least one glioma sample with hypermutation owing to the few callable sites in samples with only whole-exome data and non-hypermutant samples. All analyses were restricted to nuclei with at least 20 callable sites and at least one variant consistent with a mutation. A callable site was defined as any reference or alternative allele detected at a variant for the associated bulk DNA VCF. The C>T mutation burden metric was defined as the total number of detected C>T variants in the single-nucleus data divided by the total number of callable sites for that cell. A threshold of C>T mutation burden of high versus low was determined per platform (that is, RNA or ATAC). For each platform, a background C>T mutation burden was established by running VarTrix using hypermutant VCF from a patient on its matched non-hypermutant initial sample. Among the hypermutant samples, all nuclei with a C>T mutation burden two times the maximum non-hypermutant (initial) nuclei C>T mutation burden value were assigned as C>T high and all others as C>T low.

### Measuring the transcriptional distance between matched pairs

To quantify the transcriptional distance between matched longitudinal pairs, state-controlled pseudobulk profiles were constructed. State was controlled for by downsampling both samples to 25 nuclei per state, provided that at least 25 nuclei were present in each sample for that state. This process ensured that both samples had an equal number of nuclei and were balanced in terms of gene expression across the different cellular states. The pseudobulk profiles were generated by averaging gene expression for each gene across the selected nuclei. Finally, Euclidean distance was calculated between the two resulting vectors, which was used as the measure of transcriptional distance.

### Patient-derived GSCs

IDH-mutant GSCs T394NS and T407NS were derived from patient tumours (P61T1 and P61T2, respectively in this cohort) and expanded in orthotopic xenograft models as previously described^38^. Cells were cultured as non-adherent neurospheres in serum-free Neurobasal medium (ThermoFisher, Gibco 21103049) supplemented with 1× B-27 (ThermoFisher, 17504044), 2 mM UltraGlutamine (Lonza, BE17-605E/U1), 0.1 U ml^–1^ heparin (Sigma-Aldrich, H3149), 20 ng ml^–1^ bFGF (Miltenyi, 130-093-839), 20 ng ml^–1^ EGF (Provitro, 1325960500) and 100 U ml^–1^ penicillin–streptomycin at 37 °C, 5% CO_2_. Lines were authenticated by short tandem repeat profiling and routinely tested mycoplasma-negative.

### *IDH1* mutation validation in human MGG152 by Sanger sequencing

Genomic DNA was extracted from IDH-mutant GSC MGG152 cells^37^ using a QIAamp DNA Micro kit (Qiagen 56304). Next, 500-bp *IDH1* amplicons were amplified by using *IDH1* sense primers 5′-AATGAGCTCTATATGCCATCACTG-3′ and antisense primers 5′-TTCATACCTTGCTTAATGGGTGT-3′ with 200 ng genomic DNA template. The PCR conditions were as follows: 5 min at 95 °C, 40 cycles of 30 s at 95 °C, 30 s at 61 °C, and 30 s at 72 °C, followed by a final extension step for 7 min at 72 °C. PCR products were purified using a QIAquick PCR Purification kit (Qiagen 28104). By using both *IDH1* sense primers and antisense primers listed above, purified PCR amplicons were sequenced on Applied Biosystems 3730xL DNA Analyzers at Yale DNA Sequencing Core.

### Immunoblotting for MGG152 *CDKN2A*^–/–^ experiments

*IDH1*^*R132H*^ and *CDKN2A*^−/−^ in cells was confirmed via immunoblotting (*IDH1*^R132H^ Dianova, DIA-H09 and p16 (*CDKN2A*), Cell Signaling 80772T). β-Actin (Cell Signaling, 3700S) was used as a loading control.

### Co-culture of mouse macrophage and human IDH1-mutant GSC MGG152 cells

Primary mouse macrophages were isolated from the peritoneal cavity of stimulated C57BL/6 (wild-type) mice (3 males and 3 females, 10 weeks of age), pooled, aliquoted and cryopreserved for downstream use. On day 1 of the co-culture experiment, frozen mouse primary macrophages were rapidly thawed, and 250,000 cells were seeded in 6-well plates in DMEM medium (ThermoFisher, Gibco 11965092) with 10% FBS (ThermoFisher, Gibco A5670701) supplemented with 10 ng ml^–1^ recombinant M-CSF (BioLegend, 576406). After 12 h, macrophages were washed with MGG152 complete medium 4 times to remove residual FBS, and 450,000 dissociated MGG152 cells were seeded into the wells with macrophages for co-culture. MGG152 complete medium consisted of Neurobasal medium (ThermoFisher, Gibco 21103049) with 0.5% N-2 supplement (100×) (ThermoFisher, Gibco 17502048), 2% B-27 supplement (50×) (ThermoFisher, Gibco 17504044), 1.5% l-glutamine (Corning 25-005-CI), 0.5% antibiotic–antimycotic (Corning 30-004-CI), 20 ng ml^–1^ human FGF (PeproTech AF-100-26), 40 ng ml^–1^ human EGF (PeproTech AF-100-15), and 10 ng ml^–1^ recombinant M-CSF. The same composition for the medium was used for both monoculture and co-culture conditions. Both monoculture and co-culture cells were irradiated using Irradiator X-RAD 320 (Precision X-ray) with 2 Gy dose on each of the subsequent 3 days. After 72 h of recovery period, the cells were collected, dissociated and submitted for 10x scRNA-seq (10x Genomics).

### PDGFRA inhibitor dose–response assay

T394NS and T407NS GCSs were dissociated with accutase and seeded into ultralow-attachment 384-well plates at 500 cells per well in 25 µl complete medium, then placed on an orbital shaker for 24 h to allow uniform sphere formation. Inhibitors targeting the PDGFRA pathway, dasatinib (inhibitor of SRC, ABL and PDGFR) and CP-673451 (selective PDGFRα and PDGFRβ inhibitor) from Selleck Chemicals, were prepared as 10 mM DMSO stocks (Sigma-Aldrich) and applied in a twofold, nine-point dilution series spanning 20 µM to 40 nM; DMSO controls were included on every plate. Each condition was tested with four technical replicates and repeated in two independent experiments. Viability was measured after 3 and 6 days after treatment using CellTiter-Glo 3D (Promega) according to the manufacturer’s instructions, and luminescence was recorded on a CLARIOstar plate reader (BMG Labtech). Signals were background-subtracted and normalized to DMSO controls, and dose–response curves were fit in GraphPad Prism using a four-parameter logistic (variable-slope) model with log[inhibitor] versus normalized response to derive IC_50_ values.

### PDGFRA inhibitor treatment

Based on IC_50_ determination, 3 × 10^6^ IDH-mutant GSCs were seeded per T75 flask in 15 ml NSC medium and treated with either dasatinib or CP-673451 at 1 µM or 5 µM; DMSO served as the vehicle control with matched final concentrations across all conditions. GSCs were exposed continuously for 6 days, after which spheres were dissociated to single cells, washed in PBS and viability assessed with Trypan blue. Each condition was set up in triplicate with one replicate subjected to FACS sorting of viable cells for scRNA-seq.

### scRNA-seq using 10x Genomics

#### PDGFRA inhibitor experiments

From each condition, one replicate was FACS-enriched for viable cells and prepared using a 10x Genomics Chromium GEM-X Single Cell 3′ Gene Expression kit (v.4). Cells were loaded onto a Chromium chip with a target recovery of about 6,000–8,000 cells and run on a Chromium Controller. Individual cells were encapsulated in GEMs, in which reverse transcription and barcode incorporation occurred. Libraries were generated by disrupting the emulsions, pooling the barcoded material, amplifying cDNA, fragmenting it and adding sample indices and adapters. Library size and concentration were assessed with an Agilent High Sensitivity DNA kit on a Bioanalyzer 2100. The pooled single-cell libraries were sequenced on an Illumina NovaSeq 6000.

#### *CDKN2A*^–/–^ experiments

Parental MGG152, MGG152 non-targeting gRNA control, MGG152 *CDKN2A*^−/−^ sg1 and MGG152 *CDKN2A*^−/−^ sg3 cells were collected and dissociated into single cells. The single-cell suspension of all four cell lines was washed individually 3 times with 0.1% BSA in PBS and resuspended at 1 × 10^6^ cells per ml. Single cells were processed through a 10x Chromium 3′ Single Cell Platform using Chromium Single Cell 3′ Library, Gel Bead and Chip kits (v.3) following the manufacturers protocol. In brief, 10,000 cells were loaded into each channel of the chip to be partitioned into GEMs in a Chromium instrument, followed by barcoded reverse transcription of RNA in the droplets. This was followed by amplification, fragmentation and addition of an adaptor and sample index. Libraries from four 10x channels were pooled together (twice) and sequenced on one lane of an Illumina Next-Seq 1000/2000 sequencer with paired-end reads: read 1, 28 nucleotides; read 2, 90 nucleotides; index 1, 10 nucleotides; and index 2, 10 nucleotides.

#### Co-culture experiments

Mouse macrophage and human MGG152 cells were dissociated and processed using the 10x Chromium Single Cell 3′ v4 On-Chip Multiplexing (OCM) protocol. In total, 5,000 cells per experimental group were loaded onto the microfluidic system and all four conditions (MGG152-only, MGG152 + macrophage, MGG152-only with irradiation, and MGG152 + macrophage with irradiation) were processed together for each replicate. The resulting libraries were sequenced on an Illumina NovaSeq X.

#### IDH-mutant organoids

Three IDH-mutant astrocytoma patient-derived organoids were established from freshly resected tumour specimens obtained from the Yale University Department of Neurosurgery, following a previously described protocol^59^. Tumour tissue diagnosis was performed by neuropathological assessment and confirmed with molecular information provided by clinical whole-exome sequencing. All three organoid models maintained the *IDH1*^*R132H*^ mutation as confirmed by immunohistochemistry. For irradiation experiments, organoids were divided and allocated to either a single 10 Gy dose on day 0 or an untreated control condition, followed by a 7-day recovery period before collection. Organoids were dissociated using a Miltenyi Biotec tumour dissociation kit (Miltenyi Biotec 130-095-929) and processed through the 10x Chromium Single Cell 3′ v4 protocol. Owing to low number of organoids per condition, and therefore the low number of cells per experimental condition, all cells from each model were loaded. The resulting libraries were sequenced on an Illumina NovaSeq X. Two organoid models (GBO60 and GBO74) had sufficient myeloid cells to be analysed in Extended Data Fig. 10i.

### scRNA-seq analysis for perturbation experiments

All in vitro perturbation 10x Genomics Single Cell 3′ data were analysed using Cell Ranger (v.9.1). This version was selected because Cell Ranger v.9.0 and later are required to analyse 10x OCM libraries. For OCM libraries, the reference transcriptome was the hybrid GRCh38_and_mm10-2020-A, per the manufacturer’s instructions. For the remaining libraries, the GRCh38-2020-A reference transcriptome was used, consistent with the human glioma samples.

### Survival analyses

Survival analyses, including Kaplan–Meier plots and Cox proportional hazards models, were conducted using the R packages survminer and survival. Multiple-comparison correction was applied where indicated. Wilcoxon rank-sum and Wilcoxon signed-rank tests were two-sided unless otherwise noted.

### Statistical methods

All data analyses were conducted with R (v.4.2.0 and above), Python (v.3.6 and above) and PostgreSQL (v.14.4).

### Reporting summary

Further information on research design is available in the Nature Portfolio Reporting Summary linked to this article.

## Online content

Any methods, additional references, Nature Portfolio reporting summaries, source data, extended data, supplementary information, acknowledgements, peer review information; details of author contributions and competing interests; and statements of data and code availability are available at 10.1038/s41586-026-10612-6.

## Supplementary information

Supplementary Figs. 1–4 This file contains four Supplementary Figures and associated legends. Supplementary Fig. 1 is a heatmap of recurrence-association genetic alterations. Supplementary Fig. 2 depicts cycling malignant cells along the cellular state hierarchy. Supplementary Fig. 3 shows additional single-chromatin accessibility visualizations for malignant-cell states. Supplementary Fig. 4 includes the raw western blot images for *CDKN2A* perturbation experiments.

Reporting Summary

Supplementary Tables Supplementary Tables 1–8.

## Data Availability

The following data are available from the Gene Expression Omnibus (GEO): glioma sample gene expression count matrices from 10x Cell Ranger (v.6.1.2) (GSE326221); ATAC fragments from 10x Cell Ranger ARC (v.2.0.2) (GSE327580); and in vitro model gene expression count matrices from 10x Cell Ranger (v.9.0.1) GSE324481 for MGG152* CDKN2A*^–/–^, GSE324694 for PDGFRA inhibitors, GSE324714 for MGG152 co-culture and irradiation, and GSE324860 for organoids). Processed single-cell-state annotation and bulk processed DNA-seq data are available at Synapse (https://www.synapse.org/care_idh_mutant). The sequencing data files for the samples from SNU, Saint Joseph’s Hospital and LIH-NORLUX cohorts are available at The European Genome-phenome Archive (EGA) (EGAS50000001727). The sequencing data are available on DUOS for the MD Anderson Cancer Center cohort (DUOS-000475) and Pitié-Salpêtrière Hospital cohort (DUOS-000477). External IDH-mutant scRNA-seq and snRNA-seq datasets were downloaded from the following sources: Synapse (https://www.synapse.org/#!Synapse:syn22257780 (ref. ^8^), https://www.synapse.org/Synapse:syn60087246 (ref. ^60^) and https://www.synapse.org/#!Synapse:syn51858131 (ref. ^33^)); GEO (GSE174554 and GSE138794 (ref. ^31^), GSE205771 (ref. ^25^), GSE182109 (ref. ^32^), GSE138794 (ref. ^61^) and GSE260928 (ref. ^24^)); The Broad Institute (https://singlecell.broadinstitute.org/single_cell/study/SCP936/single-cell-multi-omics-profiling-of-human-gliomas (ref. ^7^), https://singlecell.broadinstitute.org/single_cell/study/SCP50/single-cell-rna-seq-analysis-of-astrocytoma (ref. ^4^), https://singlecell.broadinstitute.org/single_cell/study/SCP12/oligodendroglioma-intra-tumor-heterogeneity#study-download (ref. ^10^) and https://singlecell.broadinstitute.org/single_cell/study/SCP2389/programs-origins-and-niches-of-immunomodulatory-myeloid-cells-in-human-gliomas (ref. ^39^)); and Zenodo (https://zenodo.org/records/10408969)^62^. Published scRNA-seq data were also accessed from the Chinese Glioma Genome Atlas (GSE227718) and data that are not yet publicly available^26^. TCGA clinical and genomic data for the merged cohort were accessed via cBioPortal (https://www.cbioportal.org)^13^. Processed bulk DNA-seq and RNA-seq data for glioma samples that were also profiled by the GLASS consortium were accessed via Synapse (https://www.synapse.org/glass).

## Code availability

Analysis scripts for processing DNA sequencing are available at https://github.com/Kcjohnson/care-glass, and scripts for analysing single-nucleus data are available at https://github.com/Kcjohnson/care_idh_mut.
