# Supplementary material for: Acquired genetic and cell-state changes in IDH-mutant glioma progression
Source: Nature. 2026 Jun 3;655(8124):1048–59. doi: 10.1038/s41586-026-10612-6 (PMC13391360; doi:10.1038/s41586-026-10612-6)
Supplement: Supplementary file 1 — This file contains four Supplementary Figures and associated legends. Supplementary Fig. 1 is a heatmap of recurrence-association genetic alterations. Supplementary Fig. 2 depicts cycling malignant cells along the cellular state hierarchy. Supplementary Fig. 3 shows additional single-chromatin accessibility visualizations for malignant-cell states. Supplementary Fig. 4 includes the raw western blot images for CDKN2A perturbation experiments. [file 41586_2026_10612_MOESM1_ESM.pdf]

nature portfolio

https://doi.org/10.1038/s41586-026-10612-6

Supplementary information

Acquired genetic and cell-state changes in IDH-mutant glioma progression

In the format provided by the authors and unedited

Supplementary Figure 1

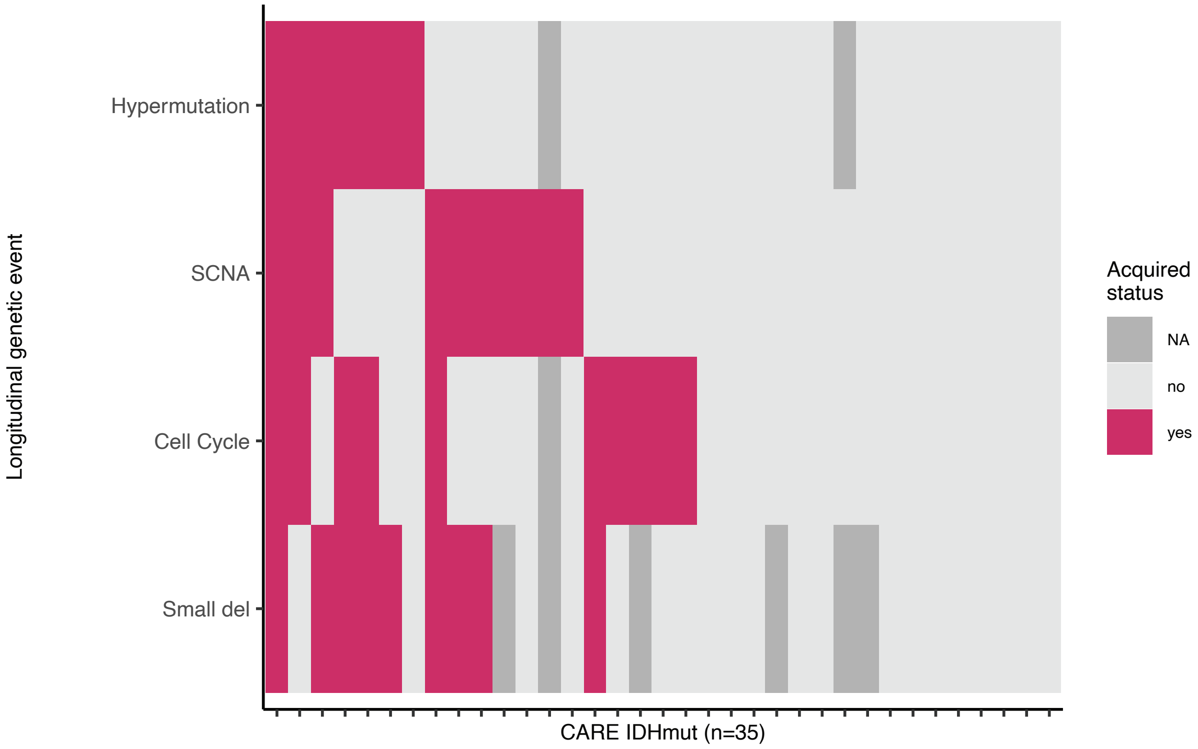

Supplementary Figure 1. Related to Figure 1. Longitudinal recurrence-assoc. genetic alterations. Heatmap displays patients (x-axis) and whether longitudinal DNA hypermutation, cell cycle alterations, small deletions, or acquired somatic copy number alterations (SCNA) were detected. NA indicates features where genetic alterations could not be determined due to missing DNA samples (e.g., normal blood or singleton tumors).

Supplementary Figure 2

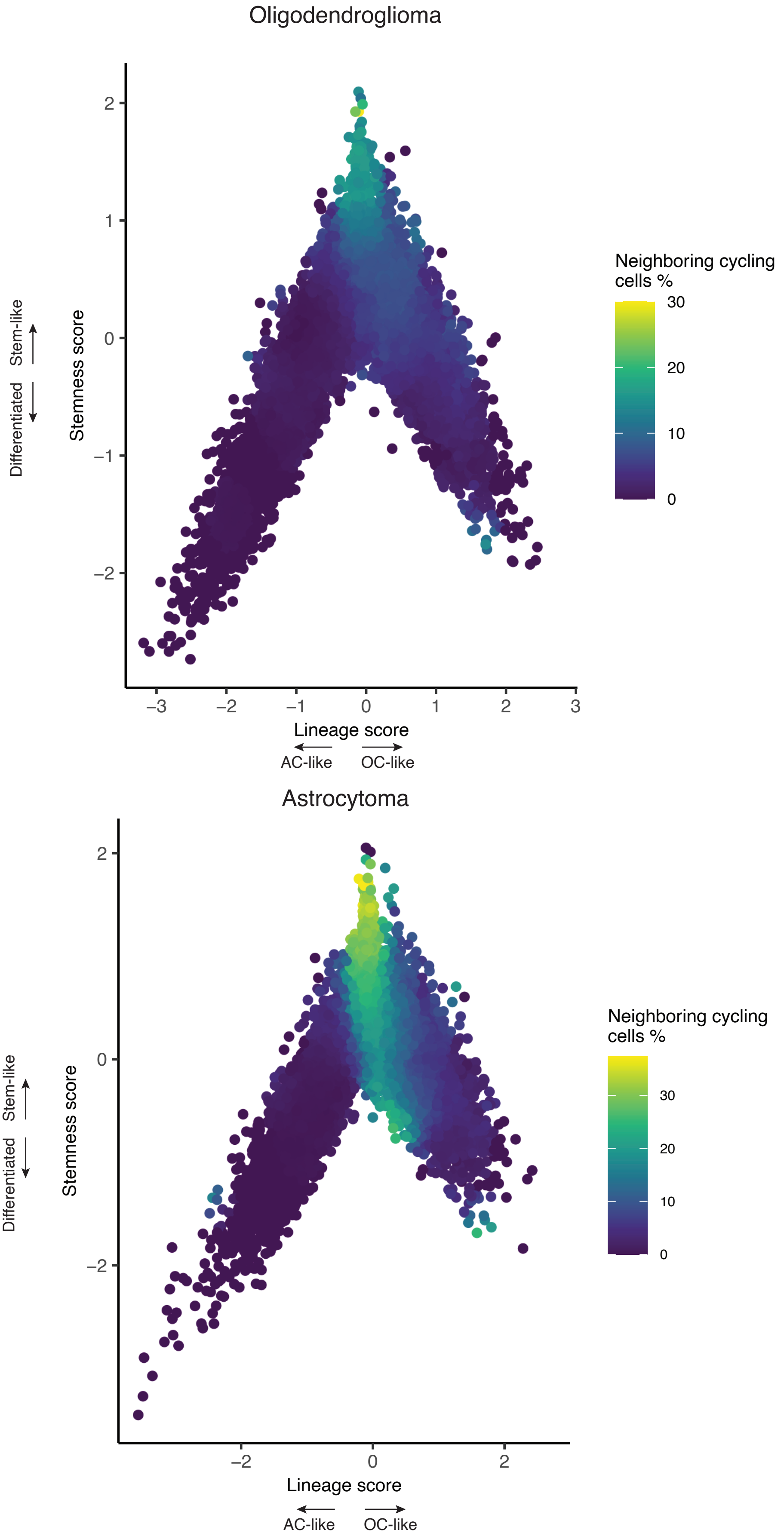

Supplementary Figure 2. Related to Figure 2. Malignant nuclei at the apex of the IDH-mutant malignant cell hierarchy are enriched for cycling nuclei. Individual nuclei are presented as circles in the IDH-mutant malignant hierarchy proposed by Tirosh et al. (PMID: 27806376). Positive stemness scores indicate greater stemness gene expression, positive lineage scores reflect oligodendrocyte lineage and negative lineage scores reflect astrocyte lineage. All nuclei within a tumor type (oligodendroglioma, top; astrocytoma, bottom) are color-coded by the fraction of neighboring nuclei (Euclidean distance of 0.3) that are cycling. Nuclei randomly down sampled to 10,000 per tumor type for visualization purposes.

Supplementary Figure 3

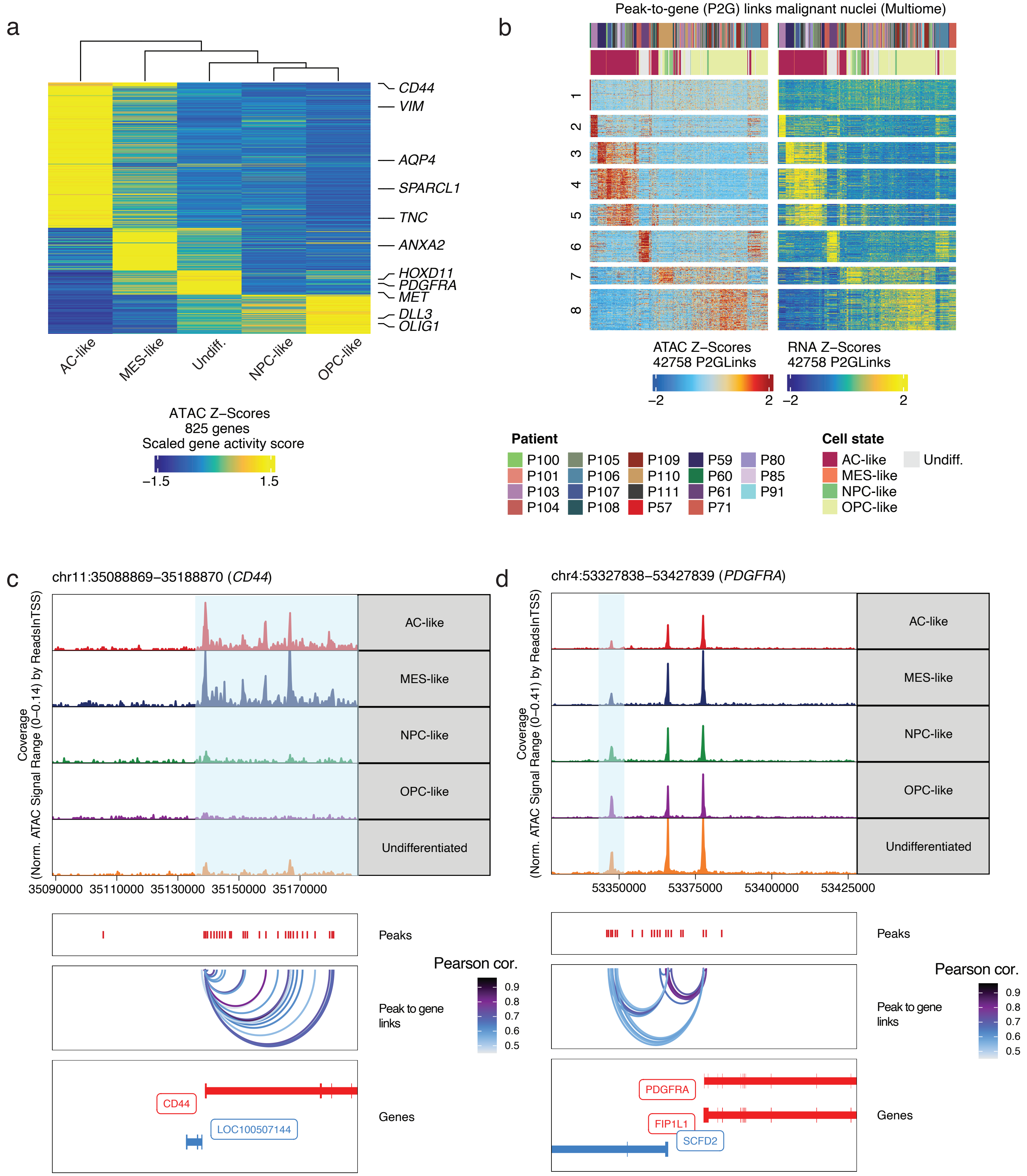

Supplementary Figure 3. Related to Figure 2. Chromatin regulation of malignant cell state-specific gene expression.
(**a**) Heatmap of snATAC gene activity scores (accessibility of a gene's regulatory elements, ArchR) for positive marker genes per cell state (Wilcoxon, FDR < 0.05, log2FC >= 1) for malignant nuclei with available snATAC data (n = 71,365 nuclei). **(b)** Peak-to-gene links with chromatin accessibility z-scores (left) and gene expression z-scores (right) annotated by patient and malignant cell state for 10x multiome samples. **(c)** Chromatin accessibility across *CD44* gene demonstrates lineage-specificity consistent with state-restricted activation of *CD44* in AC-like and MES-like malignant nuclei (top). Blue highlights chromatin areas of interest. Tracks for chromatin accessibility peaks, arcs connecting ATAC peak to genes with color intensity reflecting strength of Pearson correlation coefficient between accessibility and expression. **(d)** same as panel c but highlighting an increased in accessibility for an upstream enhancer (*PDGFRA*) for stem/progenitor states (Undifferentiated/OPC-like/NPC-like).

Supplementary Figure 4

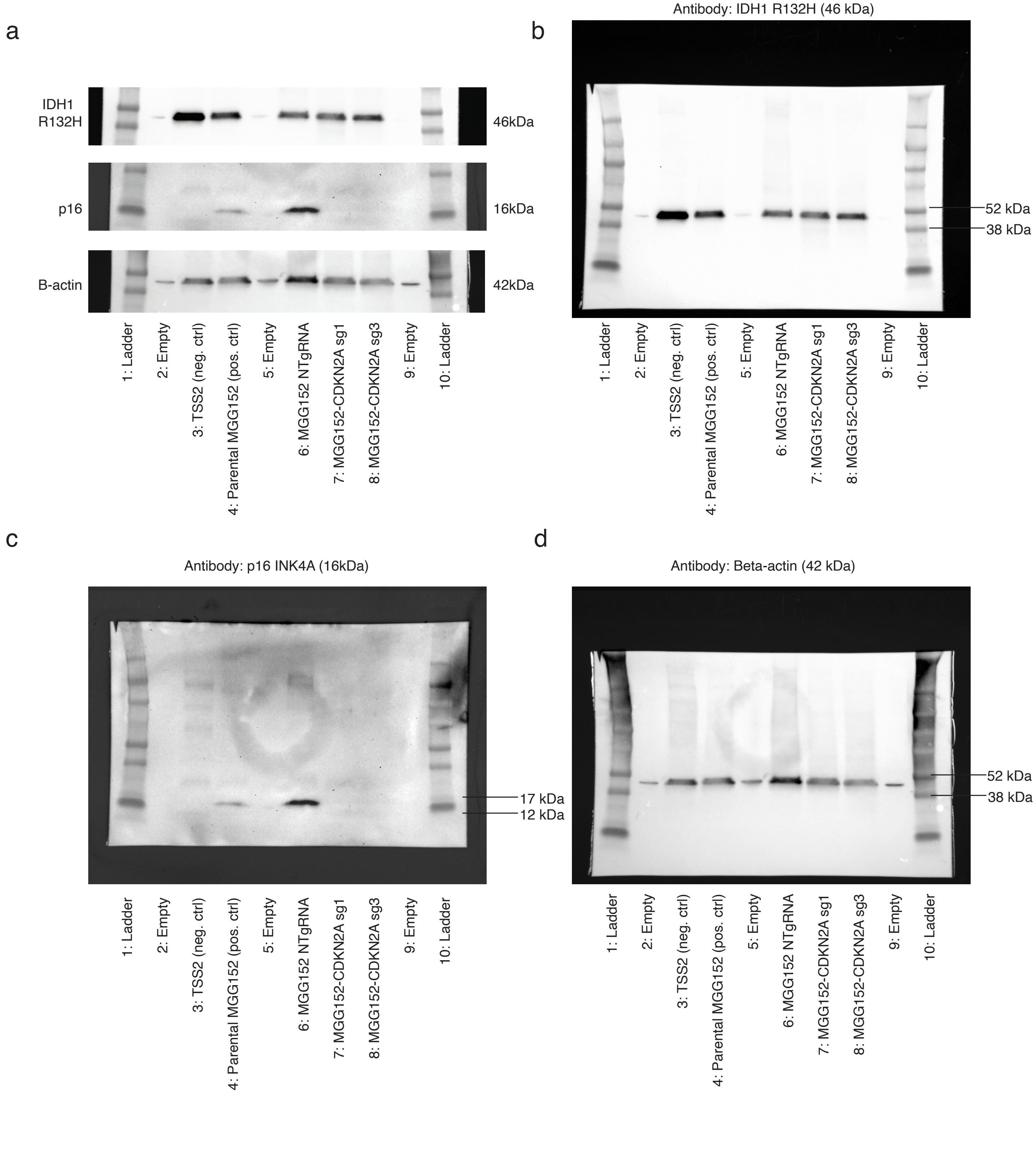

Supplementary Figure 4. **Related to Figure 4. Western blot assessment of different experimental models that assess IDH1 R132H, p16 INK4A (CDKN2A), and beta-actin protein levels**. All three proteins were assessed on the same gel. Panel **a** is the annotated composite western blot containing all three proteins, lane labels, protein names, and molecular weight markers. Panels **b-d** provides the raw images for each individual antibody. Amersham ECL Rainbow Marker full range was loaded into lanes1 and 10. The images were processed as follows: day 1) imaged p16 INK4A, after obtaining the images, the membrane was stripped for 10 minutes and incubated with IDH1 R132H primary antibody overnight, day 2) imaged IDH1 R132H, after obtaining the images, the membrane was stripped for 10 minutes and incubated with the beta-actin antibody overnight, and day 3) imaged beta-actin. Lanes 2, 5, and 9 were run as empty lanes. TSS2, an IDH1 R132H mutant cell line with *CDKN2A* deletion, served as a negative control for p16 INK4A (lane 3), Parental MGG152 (lane 4) was used as a positive control for intact p16 INK4A. Lane 6 contained MGG152 cells with a non-targeting sgRNA and lanes 7-8 include MGG152 cells with the two guides targeting *CDKN2A*.
