# Supplementary material for: Acquired genetic and cell-state changes in IDH-mutant glioma progression
Source: Nature. 2026 Jun 3;655(8124):1048–59. doi: 10.1038/s41586-026-10612-6 (PMC13391360; doi:10.1038/s41586-026-10612-6)
Supplement: Supplementary file 2 — Reporting Summary [file 41586_2026_10612_MOESM2_ESM.pdf]

nature portfolio

Corresponding author(s): Roel Verhaak

Last updated by author(s): Apr 23, 2026

Reporting Summary

Nature Portfolio wishes to improve the reproducibility of the work that we publish. This form provides structure for consistency and transparency in reporting. For further information on Nature Portfolio policies, see our Editorial Policies and the Editorial Policy Checklist.

Statistics

For all statistical analyses, confirm that the following items are present in the figure legend, table legend, main text, or Methods section.

n/a	Confirmed	
☐	☒	The exact sample size (*n*) for each experimental group/condition, given as a discrete number and unit of measurement
☐	☒	A statement on whether measurements were taken from distinct samples or whether the same sample was measured repeatedly
☐	☒	The statistical test(s) used AND whether they are one- or two-sided<br>*Only common tests should be described solely by name; describe more complex techniques in the Methods section.*
☐	☒	A description of all covariates tested
☐	☒	A description of any assumptions or corrections, such as tests of normality and adjustment for multiple comparisons
☐	☒	A full description of the statistical parameters including central tendency (e.g. means) or other basic estimates (e.g. regression coefficient) AND variation (e.g. standard deviation) or associated estimates of uncertainty (e.g. confidence intervals)
☐	☒	For null hypothesis testing, the test statistic (e.g. *F*, *t*, *r*) with confidence intervals, effect sizes, degrees of freedom and *P* value noted<br>*Give P values as exact values whenever suitable.*
☒	☐	For Bayesian analysis, information on the choice of priors and Markov chain Monte Carlo settings
☒	☐	For hierarchical and complex designs, identification of the appropriate level for tests and full reporting of outcomes
☐	☒	Estimates of effect sizes (e.g. Cohen's *d*, Pearson's *r*), indicating how they were calculated

Our web collection on statistics for biologists contains articles on many of the points above.

Software and code

Policy information about availability of computer code

Data collection	No software was used to collect data.
Data analysis	Data analysis was conducted using R (4.2.0), cellranger (6.1.2), cellranger arc (2.0.2), Seurat (4.3.0), ArchR (1.0.2), chromVARmotifs (0.2.0), harmony (1.2.0), ggpubr (0.4.0), tidyverse (1.3.1), DoubletFinder (2.0.3), infercnv (1.14.2), fgsea (1.24.0), DESeq2 (1.38.3), presto (1.0.0), msigdbr (7.5.1), GSVA (1.50.0), CopyscAT (0.40), DBI (1.2.1), survival (3.5-7), survminer (0.4.9), NMF (0.21.0), NMF clustering algorithm from Gavish et al. (https://doi.org/10.1038/s41586-023-06130-4), Python (3.6), Snakemake v5.17, BCFTools v1.9, GATK (including Mutect2) v4.1.0.0, freebayes v1.2.0, vcf2maf v1.6.16, SigProfilerExtractor tool (github.com/AlexandrovLab/SigProfilerExtractor, v1.2.1), vartrix (1.1.22). Analysis scripts for processing DNA sequencing are available at https://github.com/Kcjohnson/care-glass and scripts for analysing single-nucleus data are available at https://github.com/Kcjohnson/care_idh_mut.

For manuscripts utilizing custom algorithms or software that are central to the research but not yet described in published literature, software must be made available to editors and reviewers. We strongly encourage code deposition in a community repository (e.g. GitHub). See the Nature Portfolio guidelines for submitting code & software for further information.

Data

Policy information about availability of data

All manuscripts must include a data availability statement. This statement should provide the following information, where applicable:

- Accession codes, unique identifiers, or web links for publicly available datasets
- A description of any restrictions on data availability
- For clinical datasets or third party data, please ensure that the statement adheres to our policy

Glioma sample gene expression count matrices from 10x Cell Ranger (6.1.2) are available at Gene Expression Omnibus GSE326221 and ATAC fragments from 10x Cell Ranger ARC (2.0.2) are available at GSE327580. In vitro model gene expression count matrices from 10x Cell Ranger (9.0.1) are available at GSE324481 (MGG152 CDKN2A-/-), GSE324694 (PDGFRA inhibitors), GSE324714 (MGG152 coculture/irradiation), GSE324860 (organoids). Processed single cell state annotation, bulk processed DNA sequencing data are available on https://www.synapse.org/care_idh_mutant. The sequencing data files for the Seoul National University, Saint Joseph's Hospital, and Luxembourg Institute of Health-NORLUX cohorts are available at EGAS50000001727. The sequencing data are available on DUOS for the MD Anderson Cancer Center cohort (DUOS-000475) and Pitié-Salpêtrière Hospital cohort (DUOS-000477).

External IDH-mutant scRNAseq and snRNAseq datasets were downloaded from the following sources: Johnson et al (https://www.synapse.org/#!Synapse:syn22257780) , Wang et al (GSE174554, GSE138794), Blanco-Carmona et al (GSE205771), Abdelfattah et al (GSE182109), Wang et al (GSE138794), Johnson et al (https://www.synapse.org/Synapse:syn60087246), Chaligne et al (https://singlecell.broadinstitute.org/single_cell/study/SCP936/single-cell-multi-omics-profiling-of-human-gliomas), Venteicher et al (https://singlecell.broadinstitute.org/single_cell/study/SCP50/single-cell-rna-seq-analysis-of-astrocytoma), Tirosh et al (https://singlecell.broadinstitute.org/single_cell/study/SCP12/oligodendroglioma-intra-tumor-heterogeneity#study-download), Spitzer et al (GSE260928), Patel et al (https://www.synapse.org/#!Synapse:syn51858131) , Miller et al (https://singlecell.broadinstitute.org/single_cell/study/SCP2389/programs-origins-and-niches-of-immunomodulatory-myeloid-cells-in-human-gliomas), Ghisai et al (https://zenodo.org/records/10408969). Published scRNAseq data was also accessed from the Chinese Glioma Genome Atlas (GSE227718) as well as data that is not yet publicly available. TCGA clinical and genomic data for the merged cohort was accessed via cbiorportal.org. Processed bulk DNA and RNA sequencing data for glioma samples that were also profiled by the Glioma Longitudinal AnalySiS (GLASS) consortium were accessed via Synapse (https://www.synapse.org/glass).

Research involving human participants, their data, or biological material

Policy information about studies with human participants or human data. See also policy information about sex, gender (identity/presentation), and sexual orientation and race, ethnicity and racism.

Reporting on sex and gender	The study analyzed human IDH-mutant glioma samples. The patient sex was collected from medical records.
Reporting on race, ethnicity, or other socially relevant groupings	We did not collect information on race or ethnicity in this cohort. All patient and tumor sample characteristics are presented in Supplemental Tables 1-2
Population characteristics	The study analyzed initial and matched recurrent time point IDH-mutant glioma samples. Subject demographic information and associated clinical metadata are presented in Supplemental Tables 1-2.
Recruitment	Frozen IDH-mutant glioma samples were collected from the following tissue source sites: Saint Joseph's Hospital (Phoenix, AZ, USA), Luxembourg Institute of Health-NORLUX (LIH-NORLUX, Luxembourg), MD Anderson Cancer Center (MDACC; Houston, TX, USA), Seoul National University Hospital (Seoul, South Korea, USA), and the Pitié-Salpêtrière Hospital (Paris, France).
Ethics oversight	Frozen glioma tissue specimens with IDH1 or IDH2 gene mutation were collected with informed consent from the following tissue source sites: Saint Joseph's Hospital (Phoenix, AZ), Luxembourg Institute of Health-NORLUX (LIH-NORLUX, Luxembourg), MD Anderson Cancer Center (MDACC; Houston, TX), Seoul National University Hospital (Seoul, South Korea), and the Pitié-Salpêtrière Hospital (Paris, France). Sample collection was approved by each tissue source site's Institutional Review Board (IRB). The IRB protocol numbers of respective institutes are as follows; MDACC 2012-0441, NORLUX 201201/06, Seoul H-2004-049-1116, Pitié-Salpêtrière Hospital; 96-900, and Saint Joseph's Hospital 2020-NHSR-0084.

Note that full information on the approval of the study protocol must also be provided in the manuscript.

Field-specific reporting

Please select the one below that is the best fit for your research. If you are not sure, read the appropriate sections before making your selection.

- [x] Life sciences
- [ ] Behavioural & social sciences
- [ ] Ecological, evolutionary & environmental sciences

For a reference copy of the document with all sections, see nature.com/documents/nr-reporting-summary-flat.pdf

Life sciences study design

All studies must disclose on these points even when the disclosure is negative.

Sample size	Sample size was determined by the availability of patient-derived tumor specimens collected with at least two time points.
Data exclusions	One sample, a third time point, was excluded from analyses of malignant cells because too few malignant cells (n < 10) passed quality control in this sample.

Replication	To support our findings made in the discovery single nucleus RNA sequencing cohort, we re-analyzed publicly available IDH-mutant sn/scRNA-sequencing data (n = 139 samples), bulk The Cancer Genome Atlas (TCGA) data, and Glioma Longitudinal Analysis (GLASS) data. The reanalysis of these datasets all represent successful replication attempts because tumor grade and longitudinal changes in estimated malignant cell abundance were consistent with the present study's findings.
Randomization	No randomization was required in this study and not applicable to this study.
Blinding	Blinding was not applicable to this study because the analyses were focused on longitudinal analyses from the same patient.

Reporting for specific materials, systems and methods

We require information from authors about some types of materials, experimental systems and methods used in many studies. Here, indicate whether each material, system or method listed is relevant to your study. If you are not sure if a list item applies to your research, read the appropriate section before selecting a response.

Materials & experimental systems

n/a	Involved in the study
☒	☐ Antibodies
☐	☒ Eukaryotic cell lines
☒	☐ Palaeontology and archaeology
☒	☐ Animals and other organisms
☒	☐ Clinical data
☒	☐ Dual use research of concern
☒	☐ Plants

Methods

n/a	Involved in the study
☒	☐ ChIP-seq
☒	☐ Flow cytometry
☒	☐ MRI-based neuroimaging

Eukaryotic cell lines

Policy information about cell lines and Sex and Gender in Research

Cell line source(s)	MGG152 cell is an IDH-mutant patient-derived cell line previously reported by the Cahill and Miller labs at Massachusetts General Hospital. IDH-mutant GSCs T394NS and T407NS were derived from patient tumors (P61T1 and P61T2 at the NORLUX research institution, respectively in this cohort) and expanded in orthotopic xenograft models.
Authentication	T394NS and T407NS were authenticated by short tandem repeat profiling. MGG152 was not authenticated.
Mycoplasma contamination	MGG152, T394NS and T407NS routinely tested mycoplasma-negative.
Commonly misidentified lines (See ICLAC register)	No commonly misidentified lines were used in this study.

Plants

Seed stocks	N/A
Novel plant genotypes	N/A
Authentication	N/A
